# Structural insight for the recognition of G-quadruplex structure at human c-myc promoter sequence by flavonoid Quercetin

**DOI:** 10.1038/s41598-017-03906-3

**Published:** 2017-06-15

**Authors:** Arpita Tawani, Subodh Kumar Mishra, Amit Kumar

**Affiliations:** Centre for Biosciences and Biomedical Engineering, Indian Institute of Technology, Indore, Indore, 453552 Madhya Pradesh India

## Abstract

Small molecule ligands that could stabilize G-quadruplex structure formed at the promoter region of human c-myc oncogene will regulate its expression in cancer cells. Flavonoids, a group of naturally available small molecule, have been known for their various promising effects on human health. In present study, we have performed detailed biophysical studies for the interaction of human c-myc G-quadruplex DNA with nine representative flavonoids: Luteolin, Quercetin, Rutin, Genistein, Kaempferol, Puerarin, Hesperidin, Myricetin and Daidzein. We found by using fluorescence titration that Quercetin interacts with c-myc G-quadruplex DNA sequence Pu24T with highest affinity. This interaction was further explored by using NMR spectroscopy and we have derived the first solution structure for the complex formed between Quercetin and biologically significant c-myc promoter DNA sequence forming G-quadruplex structure. In present solution structure, Quercetin stacks at 5′ and 3′ G-tetrads of Pu24T G-quadruplex structure and stabilize it via π-π stacking interactions. Furthermore, *in vitro* studies on HeLa cells suggested that Quercetin induces apoptosis-mediated cell death and down-regulated c-myc gene expression. This study emphasizes the potential of flavonoids as a promising candidate for targeting c-myc promoter region and thus, could act as a potential anti-cancer agent.

## Introduction

G-quadruplexes are well-known secondary structures of DNA. These are non-canonical DNA structures formed by square planar arrangement of G-quartets that is stabilized by Hoogsteen hydrogen bonding^1^. In the human genome, many guanine rich sequences have potential to form G-quadruplex structures. Some of these regions are telomere^2^, regulatory regions of oncogenes such as KRAS^3^, c-myc^4^. These proto-oncogenes were found to be over expressed in 80% of cancers such as breast cancer, cervical cancer, lung cancer, etc. The promoter region of c-myc gene is composed of seven nuclease-hypersensitive elements (NHEs), of which, NHE III1 controls 80–90% transcription of c-myc gene. This 27 nucleotide sequence (5′-TGGGGAGGGTGGGGAGGGTGGGGAAGG-3′) is purine rich sequence that is also called as Pu27, has potential to form G-quadruplex structure. It is evident from one of the studies in which suppression of MYC expression was observed when Burkitt lymphoma cell lines was treated with TMPyP4, by formation of stable G-quadruplex structure. This stabilized G-quadruplex structure act as silencer element and thereby reduces the expression of c-myc gene^5^. Thus, the G-quadruplex structure in this region plays an important role as transcriptional regulator^5^.

Over the past decades, the exploration of small molecules that induces the formation of G-quadruplex structures or stabilizes them could be a potent anti-cancer agent and may act by down-regulating the oncogene expression^6^. Nature is an ample source for chemically diverse scaffolds of molecules. These naturally occurring small molecules are less toxic than the synthetic molecules and have better bio-availability^7^. Owing to the larger molecular diversity of natural compounds, research have already been initiated since long back to explore these compounds for targeting c-myc G-quadruplex structure and investigated their interaction^8^ like quindoline derivative SYUIQ-5^9^, 9-N-substituted berberine derivatives^10^, etc. Most of these compounds have planar aromatic ring system that stabilizes the G-tetrad by π- π stacking^11^. One of the major groups of naturally occurring molecules is flavonoids, that are readily available in our daily diets and have been considered as nontoxic drug candidates for anticancer therapy^12^. The common dietary flavonoids, Luteolin, Quercetin, Rutin, Genistein, Kaempferol, Puerarin, Hesperidin, Myricetin and Daidzein, have received significant attention for their anti-angiogenesis, anti-proliferative and anti-metastatic effects^13^. Earlier, one of the studies has demonstrated the interaction of Quercetin with monomeric and dimeric G-quadruplexes formed by short repeat of human telomeric sequence^14^. Recently, in 2013, a study was performed to investigate the interactions of c-myc G-quadruplex structure with a series of pyridinium side chains containing flavone derivatives. They have showed that these compounds have stronger affinity for c-myc G-quadruplex DNA over other quadruplexes and duplex DNA^15^.

**Figure 1:**
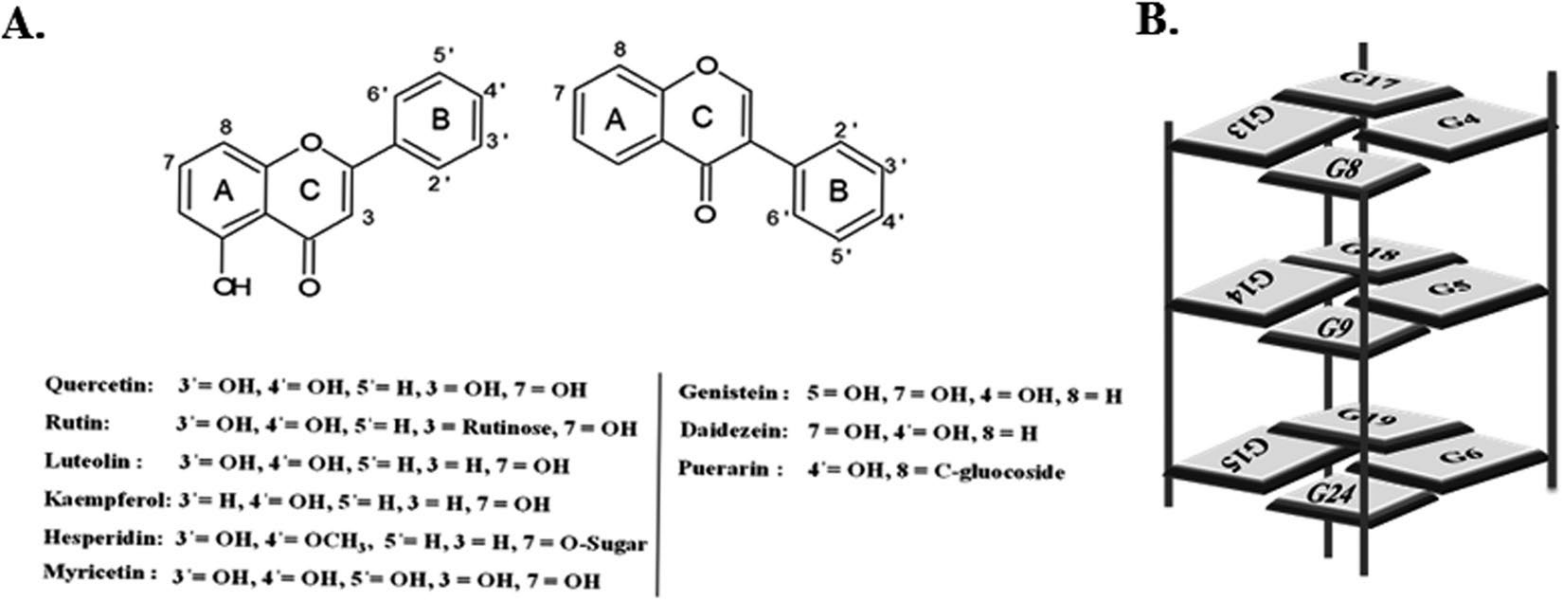
(**A**) Structure of flavonoids. Flavones, flavonols, flavanones (left side) and isoflavanols (Right side). Ring A-benzoyl system, Ring B- cinnamoyl system. (**B**) Schematic representation showing all G-tetrads involved in formingPu24T G-quadruplex DNA structure.

Albeit many studies for these effects of flavonoids, their main target for action and its mechanism still remains to be explored. Thorough structural information for mode of interaction of these flavonoids with DNA is requisite for a better understanding of the molecular basis for their therapeutic activities. Very recently we have demonstrated the interaction of Quercetin with small sequence of telomeric DNA that is TTAGGGT^16^, and, in order to get better insights about interaction of flavonoids with other G-quadruplex DNA structures, we have extended our studies with biologically significant DNA sequence in c-myc promoter region forming G-quadruplex structure. To the best of our knowledge, there is no structure available for flavonoids complexed with c-myc G-quadruplex DNA till date. In present study, we have used 24 nucleotide c-myc promoter G-quadruplex sequence Pu24T (5′-TGAGGGTGGTGAGGGTGGGGAAGG-3′) comprising of central guanine tracks of c-myc. It has been reported that this modified sequence of c-myc promoter, Pu24T provides an improved NMR spectra and also forms similar kind of G-quadruplex fold as that of biologically relevant form^17^. Thus, our present study is focused on its interaction with nine representative flavonoids Luteolin, Quercetin, Rutin, Genistein, Kaempferol, Hesperidin, Daidzein, Myricetin and Puerarin (Fig. 1). NMR studies along with other biophysical techniques, such as circular dichroism (CD), steady-state and time-resolved fluorescence spectroscopy, Isothermal titration calorimetry (ITC) were employed to investigate the binding mode of flavonoids with G-quadruplex structure formed in the human c-myc promoter region. Further, this study is centralized to get a structural basis of interaction and stabilization of intramolecular parallel G-quadruplex DNA Pu24T with most abundant naturally occurring flavonoid, Quercetin^18^. Herein, we report the first solution structure of a flavonoid Quercetin complexed with c-myc DNA Pu24T. Furthermore, *in vitro* studies were also employed to understand the cytotoxic effects of Quercetin and its subcellular localization showing its potential to down-regulate c-myc expression in human cervical carcinoma cells (HeLa cell lines).

## Results and Discussion

### Exploration of flavonoid binding to c-myc G-quadruplex DNA by steady-state and time resolved fluorescence measurements

**Figure 2:**
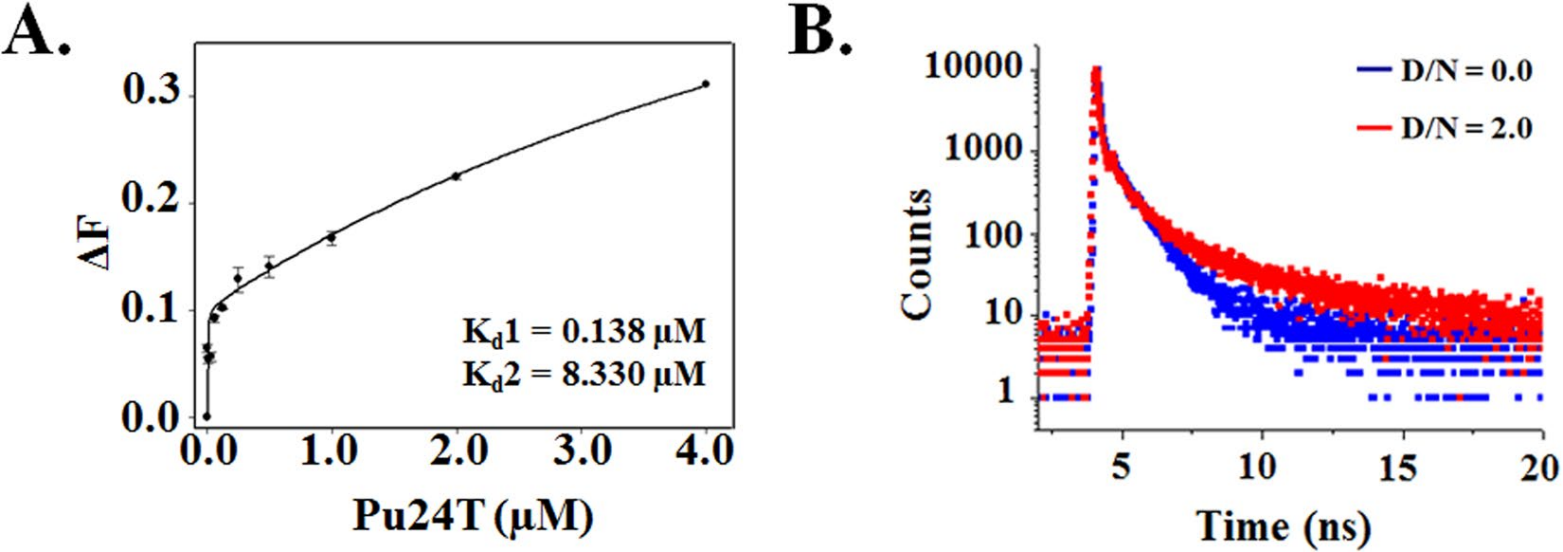
Steady state and time resolved fluorescence studies. (**A**) Fluorescence titration curve of Quercetin as a function of Pu24T concentration. Solid lines represent fit according to the ligand binding two site saturation model. Value of Binding constant(s) (K_d_) are indicated at the bottom right side of the plot. (**B**) Fluorescence life time decay curve of 40.0 µM free Quercetin (Blue) and its complex with Pu24T DNA at D/N ratio = 2.0 (Red).

**Figure 3:**
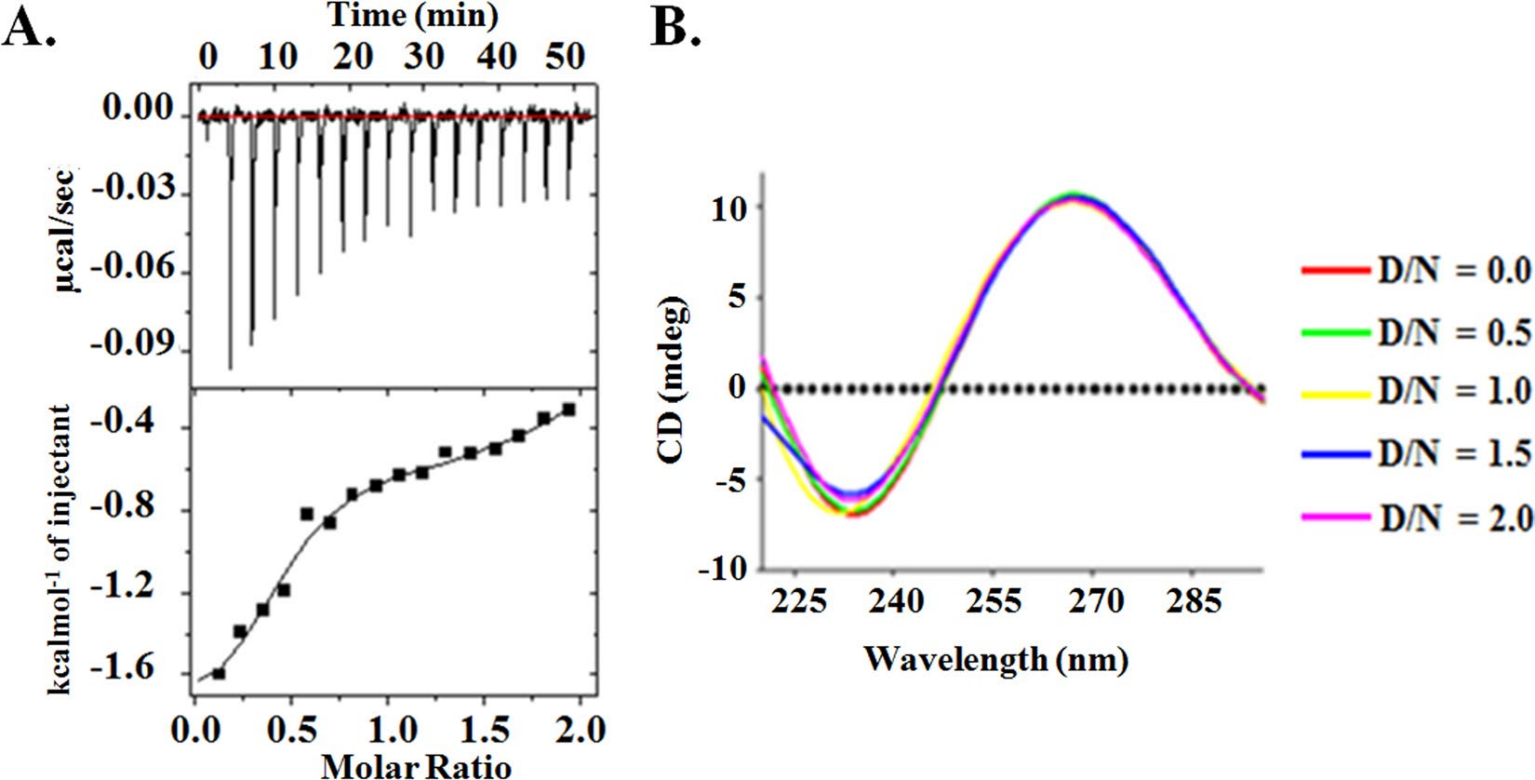
Isothermal Titration Calorimetry and Circular Dichroism studies. (**A**) ITC titration data (points) for Quercetin binding to Pu24T at 25 °C. Solid line represents the fitted data results from two site binding mode. (**B**) Circular Dichroism titration spectrum for free Pu24T (Red) and in the presence of Quercetin as a function of Quercetin concentration at D/N ratio = 0.5 to D/N ratio = 2.0. D = Quercetin; N = Pu24T.

Fluorescence titration experiments were employed to study the binding affinities of flavonoids with c-myc G-quadruplex DNA Pu24T. The fluorescence emission of all nine flavonoids was examined at the emission maximum in their unbound form. On addition of DNA, the observed changes in the fluorescence intensity depicted the binding of these flavonoids to Pu24T and generated G-quadruplex-flavonoid complex. The binding curve fitted with ligand binding two site saturation model (Fig. 2A and see Supplementary S1a) gives the binding constant values (K_d_) of these flavonoids (see Supplementary Table S1a) that strongly suggested higher affinity of Quercetin as compared to other flavonoids. A ~300 fold higher binding constant values of Quercetin for c-myc DNA as compared to duplex CT-DNA were observed (see Supplementary Fig. S2 and Table S1a) that suggested its specificity and affinity for c-myc DNA Pu24T forming G-quadruplex structure. This observed higher affinity of Quercetin amongst other flavonoids could be due to the differences in molecular structure of the flavonoids. Although all of these flavonoids have similar ring structure consisting of a heterocyclic pyrane ring (C) that links two benzene rings (A and B)^19^, the difference in pattern of substitution of the C ring along with the presence of functional groups on A and B rings causes the difference in their activity^20–22^. As in isoflavones, the B ring is attached at C3 (carbon-3) of C ring while in flavones this B ring is attached to the C2 of C ring. Moreover, the presence of hydroxyl groups enhances the DNA binding activity^23^. Further, the presence of bulky sugar rings such as in Rutin, Hesperidin, Puerarin might hinders their intercalation and binding of these flavonoids to G-quadruplex DNA^22^. Luteolin and Kaempferol lacks the hydroxyl group at 3′ of B ring while Quercetin has this hydroxyl group that might leads to better activity of Quercetin as compared to other flavonoids. Furthermore, to understand the specificity of Quercetin for c-myc promoter region with compare to other promoter regions in human genome, we have also performed the fluorescence titration experiment of Quercetin with c-kit G-quadruplex DNA. The binding constant values of Quercetin (see Supplementary Table S1b and Fig. S1b) suggested ~10 fold poor affinity than that of c-myc promoter DNA and could suggest the specificity of Quercetin for c-myc promoter region over other promoter regions. Moreover, the preferential binding of Quercetin for parallel and anti-parallel G-quartets was also assessed by performing fluorescence titration experiment with d- (T2AG3T)4 (tel7) human telomeric DNA sequence in 70 mM Na^+^ ions containing buffer. In the presence of 70 mM Na+ ions, tel7 forms anti-parallel G-quadruplex structure^24^. A ~15 fold weaker affinity was observed (see Supplementary Table S1b and Fig. S1b) for anti-parallel G-quadruplex topology as compared to Pu24T c-myc DNA forming parallel topology that suggested the preference of Quercetin for parallel G-quadruplex structures over anti-parallel G-quadruplex structure. We have also performed isothermal titration calorimetry (ITC) experiment with all flavonoids and Pu24T (Fig. 3A and see Supplementary Fig. S3 and Table S3a and b) and results are in line with fluorescence binding experiment that showed Quercetin as a lead molecule.

Since, Quercetin showed highest affinity for Pu24T amongst all the flavonoids used in this study, thus we proceeded further with Quercetin as a lead molecule and explore its binding to Pu24T in details.

The time-correlated single-photon counting (TCSPC) method was used to perform the lifetime measurement study that allowed us to determine the mode of binding of Quercetin to Pu24T. The fluorescence life time measurement is one of the useful techniques that delved the environment of fluorophore in excited state and is sentient to its interaction with DNA structure. In the unbounded form, Quercetin have three lifetimes (τ1, τ2 and τ3) and three amplitudes (β1, β2, and β3) (see Supplementary Table S2), with an average lifetime of 1.02 ps (Fig. 2B). This triexponential fluorescence decay profile indicates the presence of three different conformations in the solution probably due to the rotation of single bond between benzopyran rings to phenyl ring. On addition of Pu24T in 2:1 molar ratio of the Drug/Nucleic acid (D/N), the average lifetime of complex increased by ~25 times (see Supplementary Table S2). The best fit was achieved by triexponential fitting with slight changes in the amplitude. This attributed to the binding of Quercetin to Pu24T c-myc DNA at more than one site. In general, molecules with planar scaffold interact with G-quadruplex structure via end-stacking mode and external or groove binding mode. The lifetime property of both the binding modes is different with larger lifetime in the end-stacking mode^25^. Likely to previous study^16^, the significant changes observed in the values of both the decay components and amplitudes of Quercetin on binding with Pu24T at 2:1 ligand/Nucleic acid (D/N) ratio, specifies the interaction of flavonoids to Pu24T and the formation of the complex via end-stacking^26^.

### Isothermal calorimetry, Circular Dichroism and thermal melting studies for the interaction of Quercetin with c-myc G-quadruplex DNA

The thermogram obtained from ITC titration experiment shows that the interaction of Pu24T with Quercetin is an enthalpy-driven process that implied a much stronger interaction between them (Fig. 3A). The thermodynamic parameters such as enthalpy change ΔH, entropy change ΔS, binding constant K and stoichiometry N were calculated after fitting the data points with two binding site model using Origin 7.0 software (see Supplementary Table S3a). Our ITC data showed exothermic peaks that implies the intercalation of Quercetin due to increased π-π stacking interaction with bases of Pu24T. Also, the primary binding constant value 0.064 µM fairly shows strong interaction of Quercetin with DNA and complimented the binding constant obtained in fluorescence titration data. Further, the conformation and stability of G-quadruplex structure was also confirmed by CD experiment. In CD spectra a dominant positive peak at 260 nm and a negative peak at 240 nm represent the parallel quadruplex topology^27^. CD spectra (Fig. 3B) shows that in the presence of K^+^ ions the Pu24T-G-quadruplex DNA forms parallel G-quadruplex conformation which does not changes significantly with the addition of Quercetin. This indicated the preservation of folded G-quadruplex structure and could interpret the stability of G-quadruplex structure upon binding of Quercetin^28^.

Further, the stability of G-quadruplex structure upon binding of Quercetin was assessed by thermal melting studies. We have recorded melting curves at a wavelength of 295 nm for Pu24T G-quadruplex DNA sequences in absence and presence of Quercetin upto D/N = 2.0 ratio (see Supplementary Fig. S4). At D/N = 0.0, the melting temperature (T_m_) of Pu24T DNA was 70.0 °C. Addition of Quercetin increased the T_m_ of the Pu24T DNA to 78.0 °C and 80.0 °C at D/N = 1.0 and 2.0 respectively (see Supplementary Table S4). This 10 °C increase in T_m_ of G-quadruplex DNA is an evidence for stabilization of its structure upon addition of Quercetin.

### Comprehension of the structure of c-myc G-quadruplex DNA - Quercetin complex using NMR spectroscopy and restrained Molecular Dynamic (rMD) simulation

**Figure 4:**
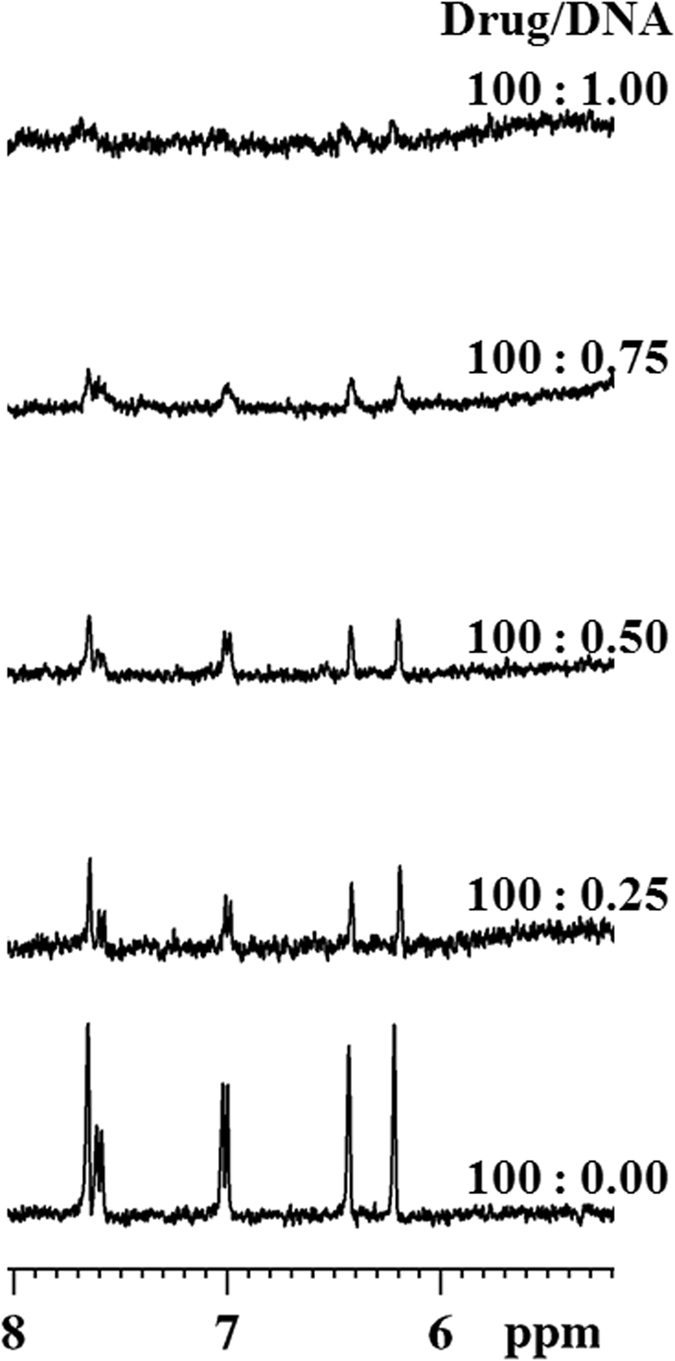
One dimensional proton spectra for Quercetin. NMR titration of 200 µM of Quercetin with increasing concentration of Pu24T from D:N = 100:0 to 100:1.

In order to determine the involvement of Quercetin protons in Drug DNA interaction, NMR titration was performed by gradual addition of Pu24T DNA into Quercetin solution. With the successive addition of Pu24T DNA solution, resonances of Quercetin protons get broadened and finally disappeared at 100: 1 D/N ratio (Fig. 4). This suggested the involvement of these protons in binding of flavonoids with Pu24T DNA.

In order to understand the structural basis of this interaction, we have performed detailed NMR studies of Drug-DNA complex that would aid in revealing the mode of binding and number of binding sites of Quercetin on Pu24T-c-myc G-quadruplex structure. Pu24T DNA sequence forms an intramolecular parallel stranded G-quadruplex structure that has a unique fold-back configuration for G24. In this structure, the G20–A21–A22–G23 forms a diagonal loop that concerted G24 into the core of G-quadruplex structure. The G-tetrad layers were bridged via formation of double-chain-reversal loops by T7, T16, and T10–G11–A12 connecting G6 to G8, G15 to G17 and G9 to G13, respectively^29^.

**Figure 5:**
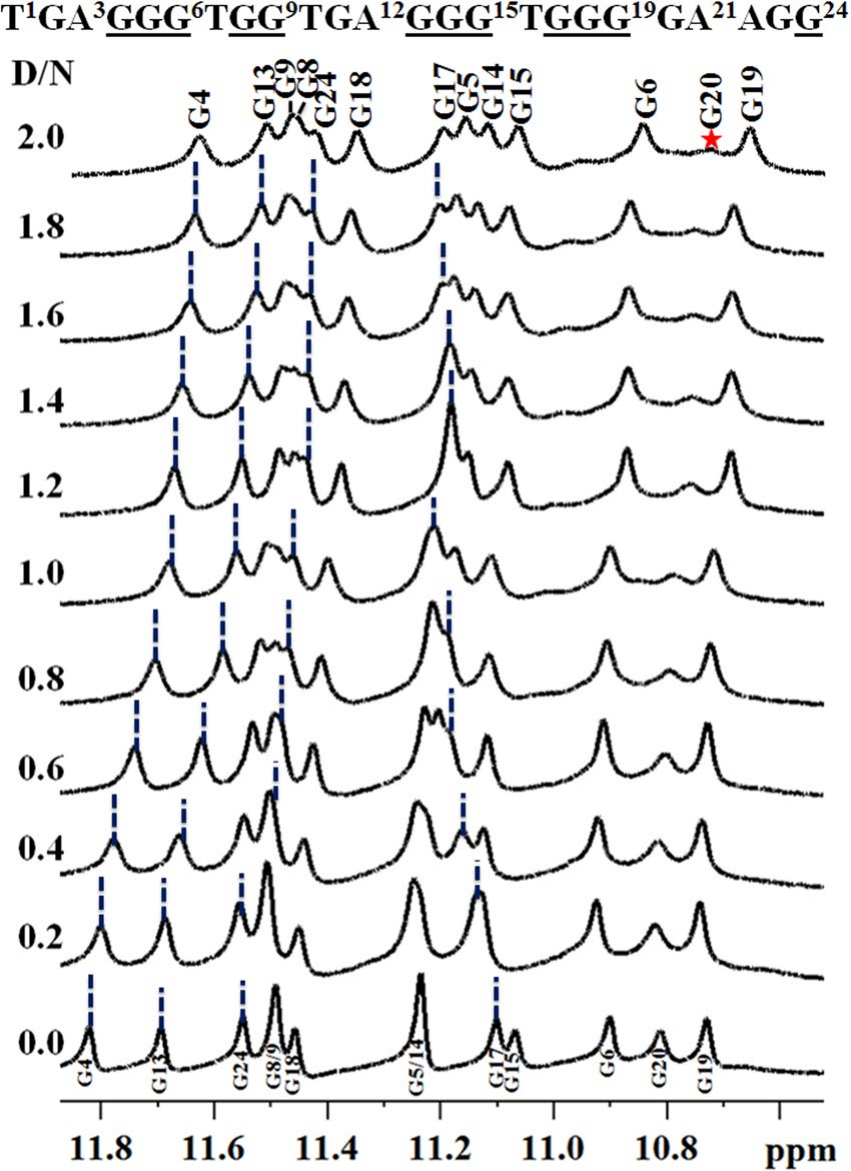
Imino region of one dimensional proton spectra for Quercetin - Pu24T complex. ^1^H NMR spectra showing the interaction of Quercetin with Pu24T monitored by imino region as a function of D/N ratio at 298 K (Changes in the chemical shift for G4, G13, G24, G17 were followed by blue dotted lines from D/N = 0.0 to D/N = 2.0 and broadening of G20 was marked as asterisk).

**Figure 6:**
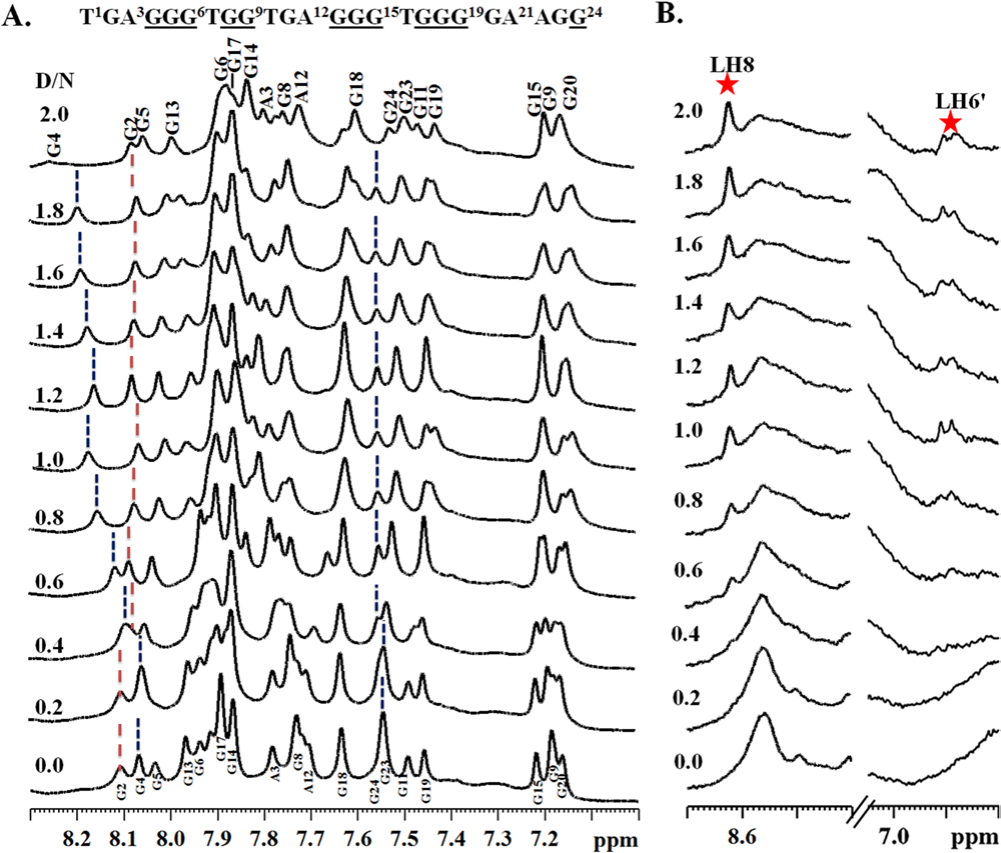
Base region of one dimensional proton spectra for Quercetin - Pu24T complex. (**A**) ^1^H NMR spectra showing the interaction of Quercetin with Pu24T monitored by base region as a function of D/N ratio at 298 K (Changes in the chemical shift for G4, G2, G24 were followed by blue dotted lines from D/N = 0.0 to D/N = 2.0. (**B**) Interaction of Quercetin with Pu24T showing proton resonances from Quercetin (LH6′ and LH8).

1-D proton NMR spectra of the Pu24T DNA were assigned based on the previous available reports^30^ and confirmed from NOE obtained from 2-D NOESY experiments. Thirteen well-resolved resonances in the imino region (10–12 ppm) (Fig. 5) of ^1^H-NMR spectra illustrated formation of well-defined G-quadruplex structure^17^. With the incremental addition of Quercetin into G-quadruplex DNA solution, the proton signals of Pu24T became broader and significant shift in resonances of imino and base regions were observed (Figs 5 and 6A). These changes started from D/N = 0.4 ratio and became prominent at D/N = 1.0 ratio and significantly clear at D/N = 2.0 ratio, thereby suggested the formation of Quercetin-Pu24T DNA complex. In the imino region of ^1^H-NMR spectra upto D/N = 2.0 (Fig. 5), the major shifts in resonances were observed for guanines that forms upper i.e. 5′ G- tetrad of Pu24T G-quadruplex DNA. G4NH and G13NH resonances showed the largest upfield shift of ~0.18 ppm while G17NH was shifted downfield by ~0.10 ppm. These shifts in G13, G17 and G4 could suggest the binding of Quercetin near to 5′ G- tetrad of Pu24T DNA. Moreover, an upfield shift of 0.08 ppm was also observed for G24NH resonance. Additionally, G20 imino proton was also significantly broadened and it gradually disappeared at D/N  = 2.0. These shifts of imino resonances could be due to π-electronic cloud^31^, and as G24 forms bottom i.e. 3′ G- tetrad and G20 base is located near to this G-tetrad, it could be inferred that Quercetin also binds at this site on Pu24T DNA. In addition to imino region, perturbations of resonances were also seen in nitrogenous base H8/H6 region of ^1^H-NMR spectra (Fig. 6A). Likewise in imino region, these changes were majorly observed for those bases that form upper and lower G-tetrad. With the incremental addition of Quercetin to Pu24T DNA solution, G4H8 proton resonance showed downfield shift of ~0.17 ppm. Further, there were 0.04 ppm upfield shift of resonances of G6H8, G17H8 protons, ~0.03 ppm for G13H8 proton and G20H8 proton shifted upfield by ~0.03 ppm. Also, the resonance of G24H8 proton became distinct with the addition of Quercetin that was initially merged at D/N = 0.0. Altogether, from the above results it could be clearly seen that perturbations in the proton resonances were observed for the guanine bases that form 5′ and 3′ G-tetrad thus corroborates the binding of Quercetin at two sites on Pu24T DNA near to these tetrads. Moreover, as depicted in Fig. 6B, the Quercetin LH6′ and LH8 proton resonates at ~6.9 ppm and ~8.63 ppm respectively, and in this region, no proton signals of free DNA were observed, therefore, it allowed us to easily monitored by 1D NMR and helps to provide the interpretation of NOE correlation in 2D NOESY spectra. Moreover, the splitting of Quercetin protons was not observed due to a rapid exchange process. Also as seen from our TSCPC studies the relaxation takes places in pico seconds (ps). The changes in the chemical environment of ligand (Quercetin) took places in very this short period of time. As the time scale for NMR experiments is in milli seconds so it was unable to capture the changes in the range of NMR time scale. However, due to overlapping with DNA proton resonances, other protons of Quercetin might not be observed that were found to be involved in the interaction as shown in Fig. 4. Besides, upfield shift was observed in imino resonances of residues forming the middle G-tetrad like G5 and G18 (Fig. 1B). However, CD titration data indicate that Quercetin does not cause the distortion of G-quadruplex structure, therefore, those shifts might be due to binding of Quercetin to neighboring G-tetrads. This is in good agreement with UV- melting data that also confirms the stability of G-quadruplex structure upon addition of Quercetin. Thus, all the above observation suggested that Quercetin does not binds in between the G-quadruplex structure and does not cause the distortion of its structure (The proton chemical shifts and individual assignments of Quercetin- Pu24T complex at D/N = 2.0 resonances are reported in Supplementary Table S5).

**Figure 7:**
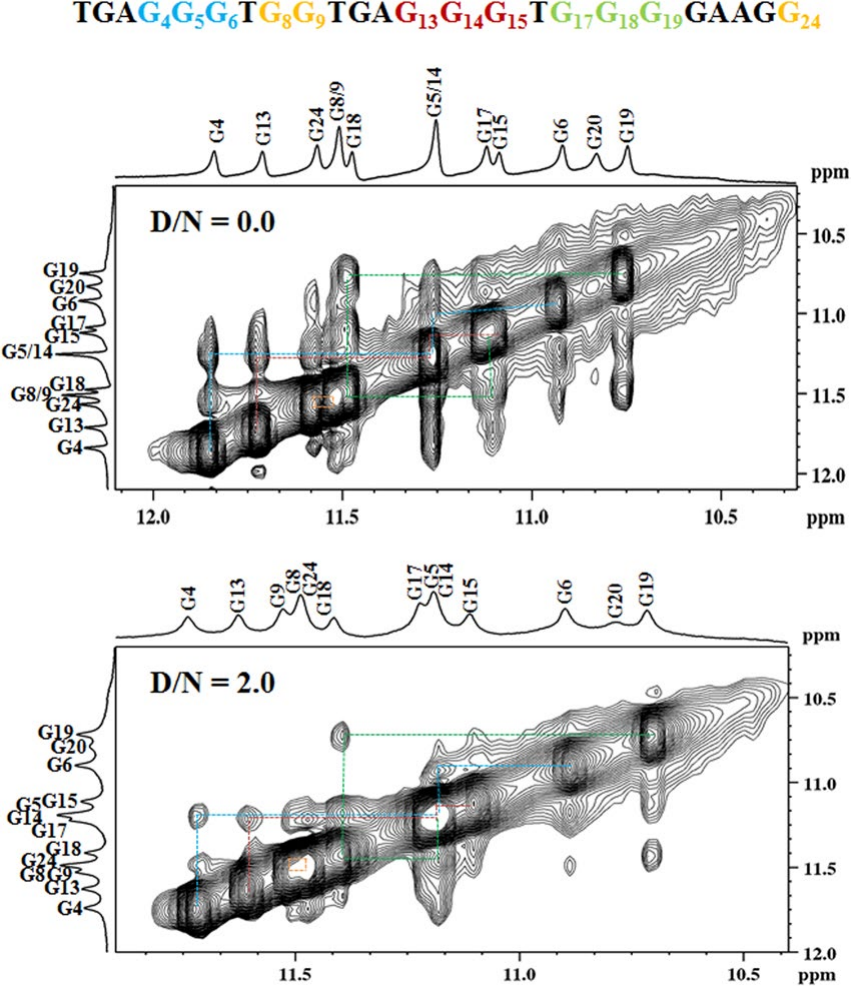
Portion of NOESY spectrum of Quercetin – Pu24T complex. Portion of NOESYspectrum of showing NH-NH NOEs between adjacent G-tetrads at 298 K at D/N ratio = 0.0 (top) and 2.0 (bottom). (Dashed lines shows the sequential connectivity).

**Figure 8:**
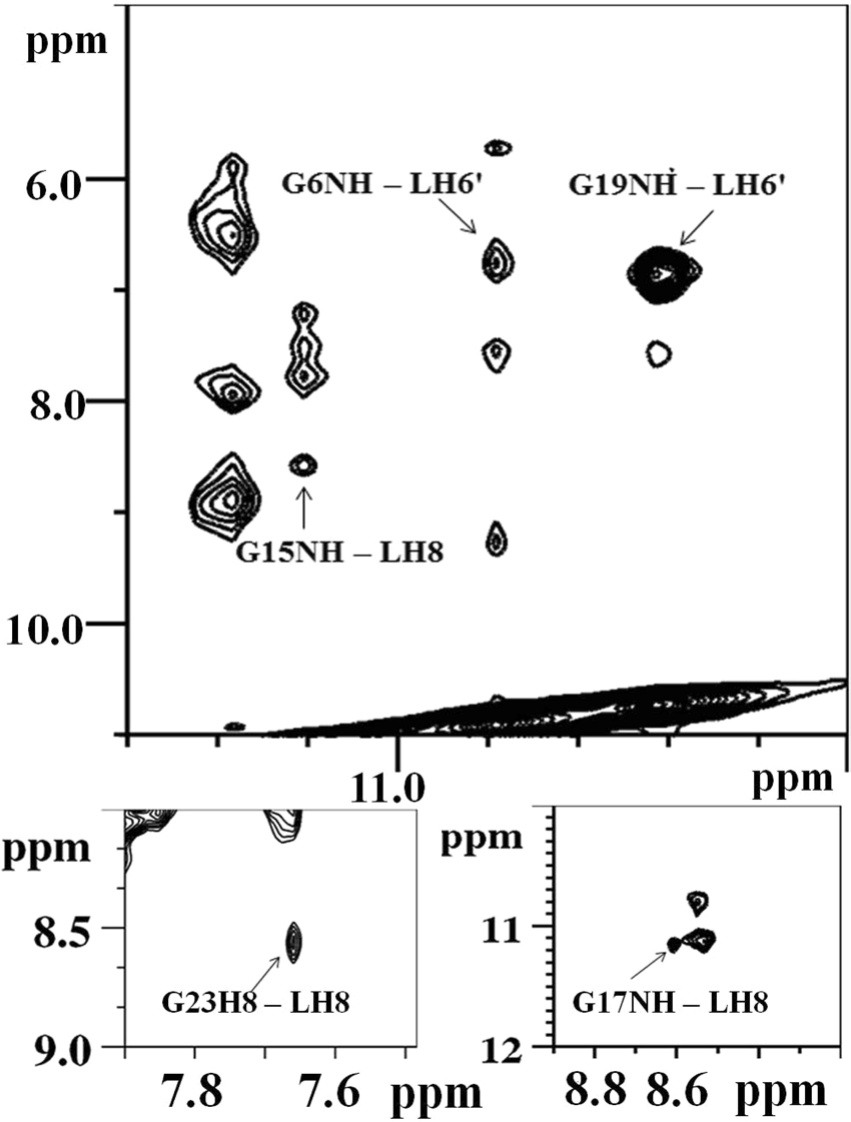
NOE cross peaks of Quercetin – Pu24T complex at D/N = 2.0. Various regions of NOESY spectrum showing intermolecular cross-peaks between Quercetin and Pu24T DNA.

**Figure 9:**
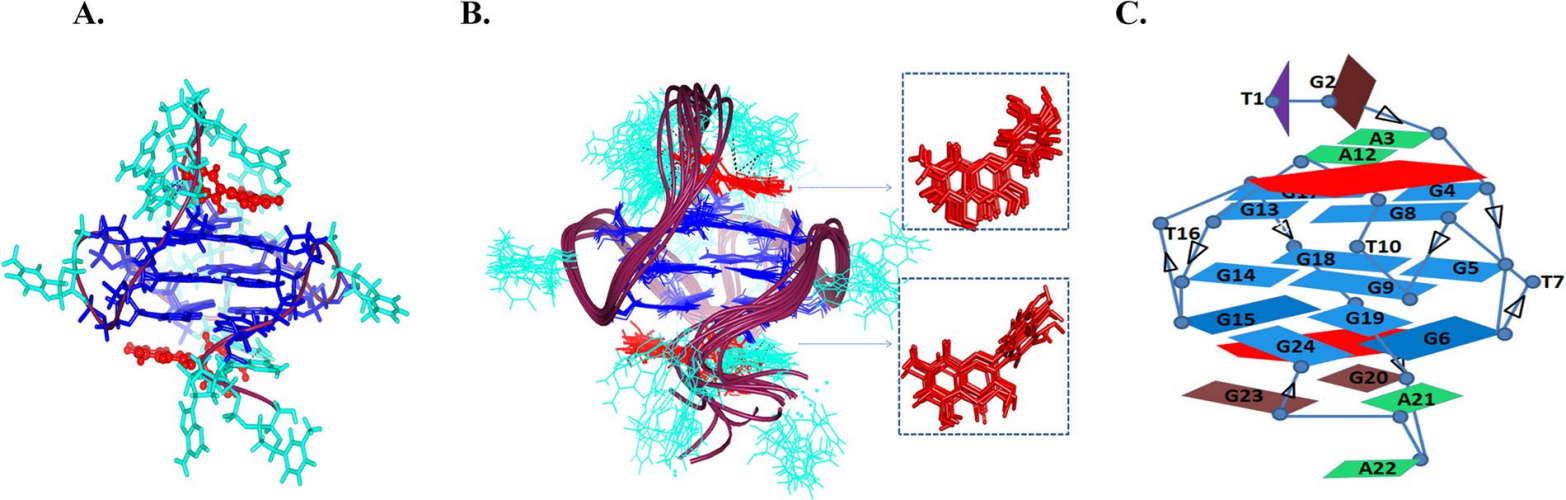
Quercetin and Pu24T complex at D/N = 2.0. (**A**) Lowest potential energy model of the complex after rMD simulation. Black dashes showing hydrogen bond formed between Quercetin (red) and Pu24T DNA. All the three G-tetrads are shown in blue and rest nucleotides are shown in cyan color, DNA backbone is shown in worm-representation. (**B**) Ensemble of ten lowest energy structures after restrained molecular dynamics simulation. (PDB Code: 2N6C) (**C**) Schematic representation showing Quercetin (red) stacking at 5′ G-tetrad and 3′ G-tetrad of Pu24T G-quadruplex DNA.

NOESY spectra were collected at various mixing time for Quercetin-Pu24T complex at different D/N ratios, that is, 0.0, 0.6, 1.0 and 2.0. The unbounded form of Pu24T displayed strong NOEs contributed by intra-nucleotide and sequential connectivities of imino protons (Fig. 7 and see Supplementary Fig. S5). This accounted for well-established stacking interaction between DNA base pairs and formation of well-defined G-quadruplex structure. Upon addition of Quercetin to DNA solution, this sequential connectivity was found to be intact which substantiates that no disruption occurs in the stacking interactions of G-quadruplex structure (Fig. 7). Interestingly, at D/N = 2.0, thirteen intermolecular NOEs were observed for Quercetin LH8 and LH6′ protons with the Pu24T protons (Supplementary Table S6 and Fig. 8) and these intermolecular NOEs can be correlated with Pu24T bases that forms both of its terminal G-tetrads, including G17, G19, G15, G6 (Fig. 8) and thus compliments the observation made from 1D NMR experiment and suggested the probable binding site of Quercetin near to these G-tetrads. Additionally, we observed a strong NOE peak for G23H8 and Quercetin LH8 proton (Fig. 8). This G23 nucleotide is located below the bottom G-tetrad (Fig. 9C), so, the observed NOE ruled out the chances of binding Quercetin in between the middle G-tetrad and thus confirms the binding of Quercetin below the bottom G-tetrad. Also as depicted in Fig. 7, the intensities of NOE cross peaks of drug DNA complex were changed as compared to free DNA that might occurred due to broadening of resonances upon binding of Quercetin. Further, with the observed intermolecular NOEs, we have calculated the structure of Pu24T G-quadruplex DNA complexed with Quercetin at D/N = 2.0 ratio via performing restrained molecular dynamics simulation on Discovery Studio Client 3.5 (Accelrys, San Diego, CA). Quercetin was placed above the 5′ G-tetrad and below the 3′ G-tetrad in an orientation that satisfied all of the NOE restraints. In a qualitative manner, the cross peak intensities were used in way such that the distances were approximately 1.8–2.5, 2.5–3.0, 3.0–3.5, 3.5–4.0, and 4.0–5.0 Å for strong intense (ss), strong (s) medium (m) and weak (w) and very weak intense (vw) peaks, respectively (see Supplementary Table S6). After the production runs of 100 ns, an ensemble of ten conformations with the lowest potential energy were superimposed (Fig. 9B) and the energy minimized model (Fig. 9A) has potential energy −18685.50 kcal/mol (see Supplementary Table S8). The distances obtained from rMD simulation results favored the experimental NOE distances (see Supplementary Table S6). The rMD simulation of Quercetin-Pu24T DNA complex showed that both the Quercetin molecules displayed dynamic behavior over the run of 100 ns but they remained bound to their respective G-tetrads. This simulation also revealed that Quercetin stacks over the G-tetrad with its A and C ring parallel and in between G17 and G13 at 5′ end while G24 and G6 at 3′ end via π-π stacking interactions (see Supplementary Figs S6 and S7). Also, the oxygen atom of 4′ OH of Quercetin forms hydrogen bond with A12 and G20 bases at 5′ end and 3′ G-tetrads respectively (see Supplementary Fig. S8.) Thus, it may be inferred that observed strong binding affinity of Quercetin with Pu24T could be due to the aromatic planar shape of Quercetin that enables it to easily end- stacks at G-tetrads of Pu24T G-quadruplex DNA and stabilize its structure (see Supplementary Fig. S9). Moreover, Pu24T G-quadruplex DNA structure has well-defined capping structures at both terminals. At 5′- end, it has TGA flanking strand; T10–G11–A12 connecting G9 to G13. The G-tetrad layers were bridged via formation of double-chain-reversal loops by T7 that connects G6 to G8, T16 connecting G15 to G17, and G20–A21–A22–G23 forms a diagonal loop that concerted G24 into the core of G-quadruplex structure. As two molecules of Quercetin end-stack at each of the terminal tetrads, therefore, it is obvious to know whether these interactions were disrupted in the complex or not. As observed in one dimensional proton spectra of Pu24T DNA, the resonances of G2, A3 H8 protons were seen as individual peak at D/N = 2.0 that were also present as same at D/N = 0.0. Further, we didn’t observe any loss of cross peak between them in NOESY spectra at D/N = 2.0 (Fig. 7). Likewise for G20 and G23 H8 protons, their resonances were observed at D/N = 2.0, but due to overlapping of peaks we couldn’t found proper sequential connectivity’s for A21 and A22 bases. In case of G11and A12, their H8 proton resonances were merged at D/N = 0.0, however these resonances were found as individual peaks at D/N = 2.0. These observations could suggest that the loop interactions were not significantly disrupted in the complex formation.

### Cytotoxic effects of Quercetin on human cervical carcinoma (HeLa) cancer cells and ***in vitro*** stabilization of c-myc G-quadruplex DNA by Quercetin

**Figure 10:**
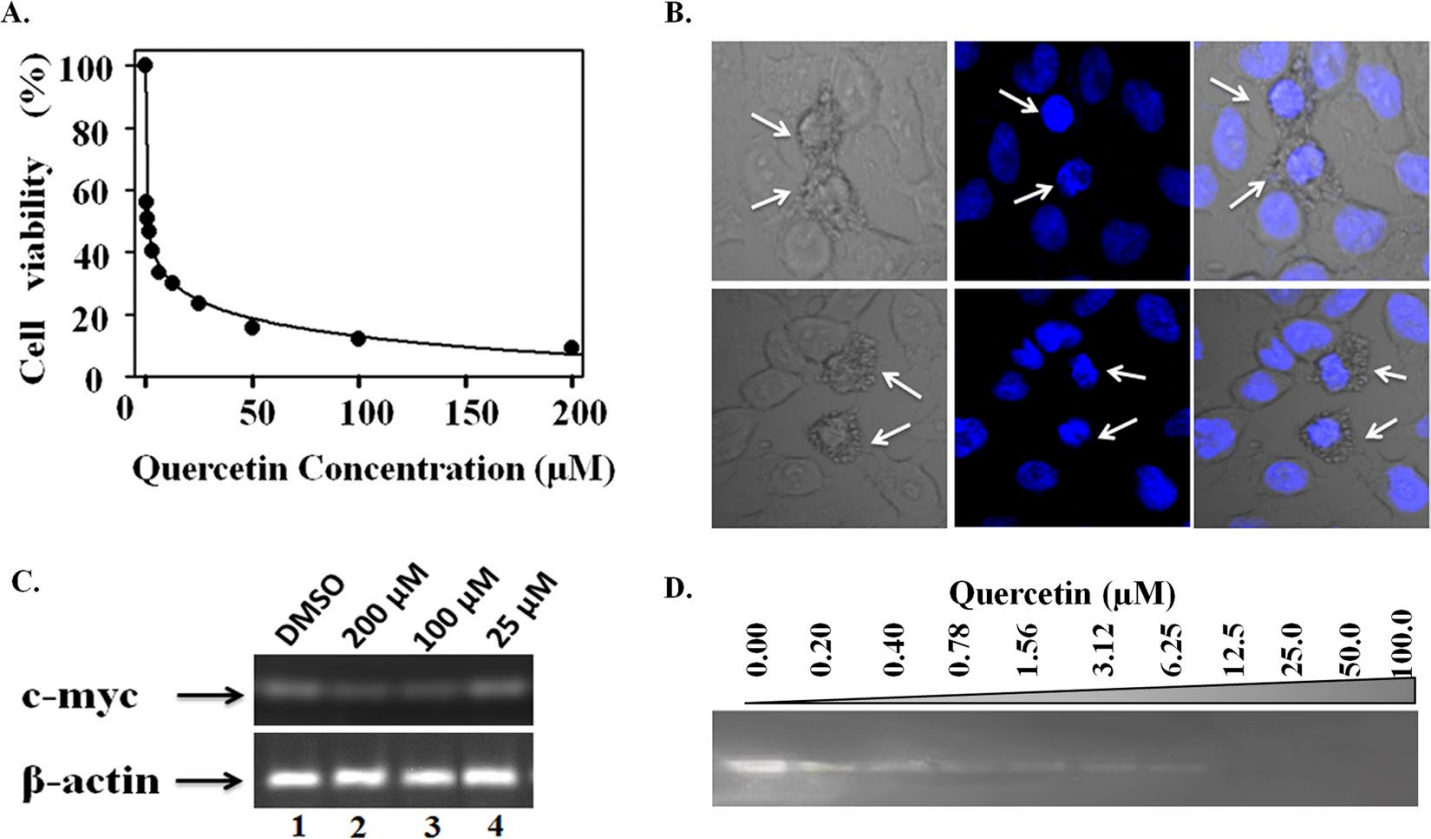
Effect of Quercetin on HeLa cell lines. (**A**) HeLa cells were exposed to Quercetin at different concentration as indicated. Cell viability was measured by MTT assay after 48 h. (**B**) Morphological changes observed under confocal microscope for Quercetin (100 µM for 4 h) treated HeLa cells followed by DAPI staining (The arrow indicates the apoptotic cells). (**C**) Representative semi-quantitative RT-PCR analysis. β-actin was used as internal control. (Full-length gel image is available at Supplementary Fig. S13). (**D**) Polymerase stop assay for determination of the effect of Quercetin on stabilization of Pu24T G-quadruplex DNA with various concentrations of Quercetin as shown.

We have performed MTT assay to determine the cytotoxicity of Quercetin in HeLa cells (Fig. 10A), that shows its potential to inhibit cell growth with an IC_50_ value of 4.0 µM. This is in agreement with previous study that shows Quercetin inhibits survival of HeLa cell with EC_50_ = 7.3 µM^32^. One of the major hallmarks of cancer is the interruption in the apoptotic pathway^33^ and anti-cancer agents induces apoptosis to overcome this interruption. In congruence with previous reports^34, 35^, we have also observed that Quercetin induces apoptosis in HeLa cells in a dose-dependent as well as in a time-dependent manner with typical apoptotic morphological changes in the cell nucleus like membrane blebbing and condensation of chromatin (Fig. 10B and see Supplementary Fig. S10). Moreover, cytotoxicity of Quercetin was also examined on normal cell line (HEK) by employing MTT assay. Our results suggested that Quercetin shows much weaker cytotoxic effect on HEK cell with over thirty-fold higher IC_50_ value of ~0.12 mM (see Supplementary Fig. S11) as compared to HeLa cell.

Previous studies demonstrated that cellular uptake of Quercetin occurs via various ways for example, in nerve cell line (SH-SY5Y), it was uptaken by mitochondria^36^, while in intestinal cell line (Caco-2), Quercetin glucoside was transported via sodium-dependent glucose transporter SGLT1^37^. In hepatocytes, it was passively diffused in cells and gets accumulated in cytoplasm as well as in nucleus; however, it was detected in cytoplasm for short time and finally gets accumulated in nucleus^38, 39^. Owing to these studies, we have also determined the localization of Quercetin inside cell nucleus. It was examined by utilizing its auto-fluorescence property and confirmed by co-localization with DAPI that showed accumulation of Quercetin within cell nucleus (see Supplemantary Fig. S10). We have also employed semi - quantitative RT-PCR to understand the effect of Quercetin on regulation of c-myc gene. This enabled us to semi-quantitate the expression of c-myc gene relative to a constitutively expressed housekeeping gene, β-actin. A significant reduction in the level of c-myc mRNA in a dose-dependent manner (Fig. 10C) was observed and as it is clearly seen that β-actin mRNA is expressed likewise in both the control as well as in treated cells, thus, the reduction of mRNA level could be specific to c-myc gene. Moreover, to confirm this reduction of c-myc gene expression is due to the stabilization of G-quadruplex structure, we have performed PCR stop assay (Fig. 10D). The observed decreased in the intensity of PCR products upon addition of Quercetin, with completely disappeared bands at 25 µM indicates that Quercetin stabilizes c-myc G-quadruplex DNA by blocking Taq Polymerase activity to amplify DNA.

Furthermore, to confirm the effect of binding of Quercetin on c-myc promoter activity, we have performed luciferase activity assay by transfecting Del-4 (wt) and Del-4-mu plasmids into HeLa cells. (“Del-4” plasmid that contains 850 bp of c-MYC promoter sequences in P1 and P2 in a luciferase reporter cassette and its variant “Del-4-mu” has specific base substitutions due to which, it is very unlikely that it forms G-quadruplex strucutre (gift from Dr. Shantanu Chowdhury)). Reduction in luciferase activity was observed in dose dependent manner for wild promoter construct, however, at the same concentrations, no trend in the change of luciferase activity was seen in cells transfected with plasmid containing mutant promoter. These results thus suggested that Quercetin inhibits the activity of c-MYC promoter by interacting and stabilizing G-quadruplex DNA structure (see Supplementary Fig. S12).

## Conclusions

In this study, we have examined the interaction of nine flavonoids with G-quadruplex DNA structure formed at promoter region of human c-myc gene. The binding properties of flavonoids with c-myc DNA gives Quercetin as a lead molecule showing highest affinity for c-myc DNA among other flavonoids and high specificity for G-quadruplex DNA structure over duplex DNA. Our study highlighted the structural aspects of binding of Quercetin to c-myc G-quadruplex DNA and we believe that this is the first report for solution structure of Quercetin - c-myc G-quadruplex complex in which Quercetin stacks at 5′ and 3′ G-tetrads of Pu24T G-quadruplex DNA structure and stabilize it via π-π stacking. Further, the biological activity of Quercetin was assessed in HeLa cells that showed its subcellular localization in nucleus. It inhibits the cell growth by inducing apoptosis and down-regulates c-myc gene expression in cancer cell. Furthermore, the stabilization of G-quadruplex structure upon binding of Quercetin was validated by PCR stop assay that shows the inhibition of *Taq Polymerase* activity.

Our study provides favorable evidences for the interaction of Quercetin with c-myc G-quadruplex DNA and its stabilization upon binding; and revealed the potential of flavonoid, Quercetin, as suitable candidate for anti-cancer therapeutics by down regulating the c-myc gene expression. The coordinates of the NMR model of the Quercetin and Pu24T complex have been deposited in the PDB as 2N6C.

## Methods

### Reagents and Cell lines

Luteolin, Quercetin, Rutin, Genistein, Kaempferol, Puerarin, Hesperidin, Myricetin, Daidzein were purchased from Sigma Aldrich Chemicals Ltd. These flavonoids were used without further purification. The solvents such as deuterium oxide, dimethyl sulphoxide (DMSO) and other reagents used for buffer preparation such as NaCl, KCl, NaH_2_PO_4_, Na_2_HPO_4_, KH_2_PO_4_ and K_2_HPO_4_ (HPLC Grade) were also purchased from Sigma Aldrich Chemicals Ltd. The stock solutions of flavonoids were prepared by dissolving them in DMSO and stored at the appropriate storage temperature.

Calf thymus DNA (CT-DNA) Pu24T-c-myc DNA (d-5′-TGAGGGTGGTGAGGGTGGGGAAGG-3′), c-kit21up: (d-5′-CGGGCGGGCGCGAGGGAGGGG-3′) and Tel7 (d-5′-T2AG3T-3′) were purchased from Sigma Aldrich Chemicals Ltd. CT-DNA solution was prepared in the sodium phosphate buffer and its concentration was measured spectrophotometerically. For quadruplex formation, 100 µM of oligomers was dissolved in phosphate buffer (10 mM (K^+^), pH 7.0) with 100 mM KCl. For anti-parallel quadruplex formation, 100 μM of Tel7 oligomers were dissolved in buffer containing 70 mM Na^+^ ions. The oligomer was annealed by heating at 90 °C for 5 mins, followed by overnight incubation at room temperature to allow gradual cooling. All the biophysical experiments were performed in the above mentioned buffer otherwise stated separately.

Human cervical carcinoma (HeLa) cell lines, Human embryonic kidney cell lines (HEK) were purchased from National Centre for Cell Science (NCCS), Pune, India. Growth media Minimum Essential Medium (MEM), Fetal bovine serum (FBS), Phosphate buffer saline (PBS), Antibiotic solution were purchased from Gibco. Cells-to-cDNA™ II Kit (Ambion) was purchased from Invitrogen. DAPI and other reagents for PCR reaction like primers, dNTPs, Taq Polymerase was also obtained from Sigma Aldrich Chemicals Ltd.

### Fluorescence Titrations

The fluorescence titration experiment was performed on Synergy™ H1 multi-mode microplate reader using 96-well microplates at 25 °C. The excitation and emission wavelengths for flavonoids were obtained by performing their absorption and fluorescence scan diluted in potassium phosphate buffer. The readings were taken at emission wavelength of 435 nm, 535 nm, 416 nm, 405 nm, 423 nm, 459 nm, 440 nm, 561 nm and 456 nm for Luteolin, Quercetin, Rutin Genistein, Kaempferol, Puerarin, Hesperidin, Myricetin and Daidzein respectively, when excited at the wavelength of 380 nm, 375 nm, 360 nm, 269 nm, 373 nm, 305 nm, 390 nm, 369 nm and 305 nm. Each sample was tested in duplicates in 75 µL reaction volume at 25 °C. The G-quadruplex DNA at a final concentration of 20 µM (4 µM for Kaempferol) was serially diluted; with the last well serve as blank (no DNA). A varied final concentration of CT-DNA (25–50 µM) was serially diluted; with the last well serve as blank (no DNA). Data were analyzed using SigmaPlot 12.0 software (Systat Software, Chicago, USA) according to the following equation and vertical lines shows standard error:

1 $${f}=\frac{{B}_{{\max }1}\times {abs}({x})}{{{k}}_{{d}1}\times {abs}({x})}+\frac{{{B}}_{{\max }2}\times {abs}({x})}{{{k}}_{{d}2}\times {abs}({x})}$$

B_max_ = maximum number of binding sites.

K_d_ = equilibrium binding constant.

### Circular Dichroism

The Circular Dichroism (CD) experiment was performed on a J-815 Spectropolarimeter (JASCO) equipped with peltier junction temperature controller. A quartz cuvette with 0.2 cm path length was used to record the spectra of samples containing 20 µM G-quadruplex and increasing concentrations of flavonoids in 100 mM KCl, 10 mM phosphate buffer (K^+^) at pH 7.0. Spectra were recorded at 0.1 nm intervals from 200 nm to 350 nm with a 1 nm-slit width and averaged over three scans. Buffer CD spectra were subtracted from the CD spectra of DNA and the Drug-DNA complex.

### Time-resolved fluorescence measurements

Time resolved fluorescence decays were collected on a Time-Correlated Single-Photon Counting (TCSPC) Spectrofluorometer (Horiba). A fixed wavelength Nano LED was used as the excitation source (ex = 375 nm), and emission was detected at a different wavelength. The fluorescence emission of Quercetin and its complex with G-quadruplex DNA were counted with a micro channel plate photo multiplier tube after passing through the monochromator and were further processed through a constant fraction discriminator (CFD), a time-to-amplitude converter (TAC) and a multi-channel analyser (MCA). The fluorescence decay was obtained and further analysed using DAS software, provided by FluoroLog-TCSPC instruments.

### Isothermal titration calorimetry experiment

The Isothermal titration calorimetry (ITC) measurements were performed at a constant temperature of 25 °C using a MicroCal^TM^ isothermal titration calorimeter iTC200 (Malvern). 2.30 μL of Quercetin was added at each step to the sample cell containing 25 µM G-quadruplex DNA. The heats of dilution were also determined by injecting same concentration of Quercetin into the same buffer. These heats of dilution were subtracted from the binding isotherm prior to fit the curve. The obtained thermogram was fitted with ‘two set of sites’ model and other thermodynamic parameters were also calculated using MicroCal Origin software.

### Nuclear Magnetic Resonance

NMR experiments were conducted on AVANCE 500 MHz BioSpin International AG, Switzerland equipped with a 5 mm broad band inverse probe. NMR data were processed, integrated and analysed on Topspin (1.3 version) software. NMR samples were referenced with 3 - (Trimethylsilyl) propionic-2, 2, 3, 3-d4 acid sodium salt (TSP). NMR studies were performed in H2O/D2O solvent at 9:1 ratio. Two-dimensional proton nuclear overhouser enhancement spectroscopy (NOESY)^40^ experiments were performed at a temperature range of 298 K with 20 ppm spectral width. Spectra were recorded at variable mixing times (tm) of 400 ms, 350 ms and 300 ms. SPARKY was used to visualize the spectra and calculate 1H-1H NOE distances, which were used to restrain Quercetin-Pu24T- G-quadruplex DNA for restrained molecular dynamic simulation studies.

### Restrained Molecular Dynamics studies

The structure of G-quadruplex Pu24T (PDB code: 2MGN^30^) was taken as the starting model and the required replacements, addition of residues were performed on Discovery studio 3.5 (Accelrys Inc., USA). G-quadruplex- the Quercetin complex was built by placing ligand above the 5′ G-tetrad and below the 3′ G-tetrad with orientations obtained from NOE experimental data. A set of NOE distances was introduced as restraints with a force constant of −10 kcal/mol/Å^2^. The drug-quadruplex system was typed in charmM forcefield^41^ and solvated with periodic TIP3P^42^ orthorhombic water box containing 1720 water molecules. After minimization of complex, the conformations with the lowest potential energy were obtained by subjecting the quadruplex-ligand complex to simulated annealing restrained molecular dynamics with the whole set of NOE restraints. Standard dynamic cascade runs were performed on the complex in which the system was heated to 700 K followed by equilibration under constant pressure for 1 ps. The production was done at 300 K for 100 ns in an NPT ensemble and long range electrostatics were treated with the Particle Mesh Ewald (PME) method^43^ with a 14 Å cut-off radius counted the non-bonded distances. To constrain the motion of H-bonds, the SHAKE algorithm^44^ was applied during the whole simulation runs.

### PCR Stop assay

The assay was performed by employing modified protocol of previous study^45^ using a test oligonucleotide c-myc Pu24T: d-(5′-TGAGGGTGGTGAGGGTGGGGAAGG-3′) and a complementary oligonucleotide (RevPu24T): d-(5′-TTCTCGTCCTTCCCCA-3′). Assay reactions were performed in a final volume of 25 μL reaction mixture containing 10 mM Tris buffer, 50 mM KCl, 10.0 pmol of each oligonucleotide, 2.5 units of Taq polymerase and the varied concentration of Quercetin from 0.00 μM to 100.00 μM. Reaction mixtures were incubated in Mastercycler Nexus Gradient (Eppendorf) with the following cycling conditions: 94 °C for 2 min, followed by 30 cycles of 94 °C for 30 s, 58 °C for 30 s, and 72 °C for 30 s. Amplified products were resolved on a 3% agarose gel in 1X TBE and stained with EtBr. Gel Image was analyzed on ImageQuant LAS 4000 (GE Healthcare).

### MTT assay

The cytotoxic effects of Quercetin was evaluated by performing MTT (3-(4, 5-dimethylthiazol-2-yl)-2,5 diphenyltetrazolium bromide dye) assay. HeLa cells (5.0 × 10^3^ cells/well) were seeded in a 96-well culture plate in triplicate and allowed to grow in MEM complete medium. Cells were treated with different concentrations (200 *μ*M to 0.2 *μ*M) of Quercetin with DMSO in control sample for 48 h at 37 °C, 5% CO2. After incubation, 10 *μ*L of MTT (5 mg/mL in PBS) was added to each well and incubated for additional four hours at 37 °C to allow intracellular reduction of the soluble yellow MTT to insoluble purple formazan crystals. These crystals were dissolved by adding 100 *μ*L of DMSO and absorbance was read at 570 nm using a microplate reader (Synergy™ H1 multi-mode microplate reader). Concentration of Quercetin causing 50% reduction of cell viability was inhibitory concentration (IC_50_ value), was determined by the using formula:

2 $$ \% \,{\boldsymbol{inhibition}}=\,\frac{{\boldsymbol{Control}}\,{\boldsymbol{abs}}-{\boldsymbol{Sample}}\,{\boldsymbol{abs}}}{{\boldsymbol{Control}}\,{\boldsymbol{abs}}}\times {\bf{100}}$$

### Confocal microscopy for localization of Quercetin in cells

HeLa cells were grown on glass cover slip treated with 100 μM Quercetin for 4 h. Cells were fixed with 10% formalin and cover slip was mounted on glass slide. Simultaneously, one of the set of cells was processed for DAPI staining for 20 mins at room temperature in dark. Afterwards, the auto fluorescence of Quercetin in HeLa cells was monitored under confocal laser scanning microscope (Olympus 1 × 83, Japan) and data were analysed using Olympus Fluoview 4.2a software. Control cells were treated with DMSO and they do not exhibit auto-fluorescence, therefore they were not shown here. At least 10 fields per slide and three independent sets were examined.

### Semi-quantitative RT PCR analysis

HeLa cells were grown in T-25 tissue culture flask and incubated with various concentrations (200.0, 100.0, 50.0 and 25.0 μM) of Quercetin for 24 h at 37 °C in humidified 5% CO_2_ incubator. Total RNA was prepared from treated and control cells and cDNA was prepared using Cells-to-cDNA™ II Kit (Ambion) according to the manufacturer’s protocol. Reverse transcriptase reaction was performed on Mastercycler Nexus Gradient (Eppendorf). The thermal cycling condition was programmed as 45 min at 45 °C, 10 min at 95 °C for one single cycle. Semi – quantitative PCR was performed using gene specific primers with the following sequences: c-MYC (forward): 5′-CTTCTCTCCGTCCTCGGATTCT-3′; c-MYC (reverse): 5′-GAAGGTGATCCAGACTCTGACCTT-3′; β-actin (forward): 5′- GAGCTACGAGCTGCCTGAC-3′; β - actin (reverse): 5′-AGCACTGTGTTGGCGTACAG-3′.

## Electronic supplementary material

Supplementary Information

## References

[1] Sen D, Gilbert W (1988). Formation of parallel four-stranded complexes by guanine-rich motifs in DNA and its implications for meiosis. Nature.

[2] Wang Y, Patel DJ (1993). Solution structure of the human telomeric repeat d[AG3(T2AG3)3] G-tetraplex. Structure.

[3] Cogoi S, Xodo LE (2006). G-quadruplex formation within the promoter of the KRAS proto-oncogene and its effect on transcription. Nucleic Acids Res.

[4] Yang D, Hurley LH (2006). Structure of the biologically relevant G-quadruplex in the c-MYC promoter. Nucleos. Nucleot. Nucl.

[5] Siddiqui-Jain A, Grand CL, Bearss DJ, Hurley LH (2002). Direct evidence for a G-quadruplex in a promoter region and its targeting with a small molecule to repress c-MYC transcription. Proc. Natl. Acad. Sci. USA.

[6] Nasiri HR (2014). Targeting a c-MYC G-quadruplex DNA with a fragment library. Chem. Commun..

[7] Neidle S, Thurston DE (2005). Chemical approaches to the discovery and development of cancer therapies. Nat. Rev. Cancer.

[8] Efferth T, Li PC, Konkimalla VS, Kaina B (2007). From traditional Chinese medicine to rational cancer therapy. Trends Mol. Med..

[9] Liu JN (2007). Inhibition of myc promoter and telomerase activity and induction of delayed apoptosis by SYUIQ-5, a novel G-quadruplex interactive agent in leukemia cells. Leukemia.

[10] Ma Y (2008). 9-N-Substituted berberine derivatives: stabilization of G-quadruplex DNA and down-regulation of oncogene c-myc. Bioorg. Med. Chem..

[11] Fedoroff OY (1998). NMR-Based model of a telomerase-inhibiting compound bound to G-quadruplex DNA. Biochemistry.

[12] Carini JP, Klamt F, Bassani VL (2014). Flavonoids from Achyrocline satureioides: promising biomolecules for anticancer therapy. RSC Adv.

[13] Kuntz S, Wenzel U, Daniel H (1999). Comparative analysis of the effects of flavonoids on proliferation, cytotoxicity, and apoptosis in human colon cancer cell lines. Eur. J. Nutr..

[14] Sun H (2006). Spectroscopic studies of the interaction between quercetin and G-quadruplex DNA. Bioorg. Med. Chem. Lett..

[15] Yang H (2013). Structure-based design of flavone derivatives as c-myc oncogene down-regulators. Eur. J. Pharm. Sci..

[16] Tawani A, Kumar A (2015). Structural Insight into the interaction of Flavonoids with Human Telomeric Sequence. Sci. Rep..

[17] Phan AT, Kuryavyi V, Gaw HY, Patel DJ (2005). Small-molecule interaction with a five-guanine-tract G-quadruplex structure from the human MYC promoter. Nat. Chem. Biol..

[18] Burda S, Oleszek W (2001). Antioxidant and antiradical activities of flavonoids. J. Agric. Food. Chem..

[19] Kumar S, Pandey AK (2013). Chemistry and Biological Activities of Flavonoids: An Overview. Scientific World J.

[20] Rice-Evans CA, Miller NJ, Bolwell PG, Bramley PM, Pridham JB (1995). The relative antioxidant activities of plant-derived polyphenolic flavonoids. Free Radic. Res..

[21] Sroka Z (2005). Antioxidative and antiradical properties of plant phenolics. Z. Naturforsch. C Biol. Sci.

[22] Wang Z (2008). Evaluation of Flavonoids Binding to DNA Duplexes by Electrospray Ionization Mass Spectrometry. J. Am. Soc. Mass Spectrom.

[23] Ragazzon PA, Iley J, Missailidis S (2009). Structure-activity studies of the binding of the flavonoid scaffold to DNA. Anticancer Res..

[24] Balagurumoorthy P, Brahmachari SK (1994). Structure and stability of human telomeric sequence. J. Biol. Chem..

[25] Wei C, Wang J, Zhang M (2010). Spectroscopic study on the binding of porphyrins to (G(4)T(4)G(4))4 parallel G-quadruplex. Biophys. Chem..

[26] Tysoe SA, Morgan RJ, Baker AD, Strekas TC (1993). Spectroscopic investigation of differential binding modes of.DELTA - and LAMBDA -Ru(bpy)2(ppz)2+ with calf thymus DNA. J. Phys. Chem..

[27] Kypr J, Kejnovská I, Renčiuk D, Vorlíčková M (2009). Circular dichroism and conformational polymorphism of DNA. Nucleic Acids Res.

[28] Ranjan N, Andreasen KF, Kumar S, Hyde-Volpe D, Arya DP (2010). Aminoglycoside binding to Oxytricha nova telomeric DNA. Biochemistry.

[29] Ma DL (2012). Discovery of a natural product-like c-myc G-quadruplex DNA groove-binder by molecular docking. PloS one.

[30] Chung WJ, Heddi B, Hamon F, Teulade-Fichou MP, Phan AT (2014). Solution structure of a G-quadruplex bound to the bisquinolinium compound Phen-DC(3). Angew. Chem. Int. Ed. Engl..

[31] Simonsson T, Pecinka P, Kubista M (1998). DNA tetraplex formation in the control region of c-myc. Nucleic Acids Res.

[32] Durgo K, Vukovic L, Rusak G, Osmak M, Colic JF (2009). Cytotoxic and apoptotic effect of structurally similar flavonoids on parental and drug-resistant cells of a human cervical carcinoma. Food Technol. Biotechnol..

[33] Hanahan D, Weinberg RA (2000). The hallmarks of cancer. Cell.

[34] Zhang W, Zhang F (2009). Effects of quercetin on proliferation, apoptosis, adhesion and migration, and invasion of HeLa cells. Eur. J. Gynaecol. Oncol..

[35] Wang Y, Zhang W, Lv Q, Zhang J, Zhu D (2016). The critical role of quercetin in autophagy and apoptosis in HeLa cells. Tumor Biol..

[36] Bandaruk Y, Mukai R, Terao J (2014). Cellular uptake of quercetin and luteolin and their effects on monoamine oxidase-A in human neuroblastoma SH-SY5Y cells. Toxicol. Rep.

[37] Walgren RA, Lin JT, Kinne RK, Walle T (2000). Cellular uptake of dietary flavonoid quercetin 4′-beta-glucoside by sodium-dependent glucose transporter SGLT1. J. Pharmacol. Exp. Ther..

[38] Mukai R, Shirai Y, Saito N (2009). Yoshida, K.-i. & Ashida, H. Subcellular localization of flavonol aglycone in hepatocytes visualized by confocal laser scanning fluorescence microscope. Cytotechnology.

[39] Gonzales GB (2015). Review on the Use of Cell Cultures to Study Metabolism, Transport, and Accumulation of Flavonoids: From Mono-Cultures to Co-Culture Systems. Compr. Rev. Food Sci. Food Saf..

[40] Jeener J, Meier BH, Bachmann P, Ernst RR (1979). Investigation of exchange processes by two‐dimensional NMR spectroscopy. J. Comput. Chem..

[41] Brooks BR (2009). CHARMM: the biomolecular simulation program. J. Comput. Chem..

[42] Jorgensen WL, Chandrasekhar J, Madura JD, Impey RW, Klein ML (1983). Comparison of simple potential functions for simulating liquid water. J. Chem. Phys..

[43] Darden T, York D, Pedersen L (1993). Particle mesh Ewald: An N⋅log(N) method for Ewald sums in large systems. J. Chem. Phys..

[44] Ryckaert J-P, Ciccotti G, Berendsen HJC (1977). Numerical integration of the cartesian equations of motion of a system with constraints: molecular dynamics of n-alkanes. J. Comput. Phys..

[45] Lemarteleur T (2004). Stabilization of the c-myc gene promoter quadruplex by specific ligands’ inhibitors of telomerase. Biochem. Biophys. Res. Commun..

