# Supplementary material for: Structural insight for the recognition of G-quadruplex structure at human c-myc promoter sequence by flavonoid Quercetin
Source: Sci Rep. 2017 Jun 15;7:3600. doi: 10.1038/s41598-017-03906-3 (PMC5472631; doi:10.1038/s41598-017-03906-3)

Supplementary Information

Structural insight for the recognition of G-quadruplex structure at human c-myc promoter sequence by flavonoid Quercetin.

Arpita Tawani[a], Subodh Kumar Mishra[a] & Amit Kumar[a*]

[a] Centre for Biosciences and Biomedical Engineering, Indian Institute of Technology, Indore.

Indore, 453552, Madhya Pradesh, India

E-mail: amitk@iiti.ac.in

Index

SI. Supplementary Tables S4-S9

Table S1a. Binding constant values as obtained from flourescence titration experiment for all flavonoids with *c-myc* DNA and CT- DNA. S4

Table S1b. Binding constant values as obtained from flourescence titration experiment for Quercetin with Tel7 and c-kit G-quadruplex DNA. S4

Table S2. Life time fluorescence decay parameters for Quercetin and its D/N = 2:1 complex with *c-myc* DNA at 298 K. S5

Table S3a. Thermodynamic and energetics data for the interaction Quercetin with *c-myc* DNA as obtained from ITC experiment. S5

Table S3b. Thermodynamic and energetics data for the interaction all flavonoids with *c-myc* DNA as obtained from ITC experiment. S5

Table S4. The melting temperature values of Pu24T G-quadruplex DNA in absence and presence of Quercetin.0 S6

Table S5. Proton chemical shift table for Pu24T-*c-myc* DNA and Quercetin complex at D/N = 2.0 S7

Table S6. Relative intensity of intermolecular NOE connectivity between Pu24T-*c-myc* DNA and Quercetin in the complex at D/N = 2.0 from NOESY spectra at 298K. S8

Table S7. Statistics of the solution structure of Quercetin- Pu24T-*c-myc* DNA complex. S9

Table S8. Energy terms (kcal mol^{-1}) for starting structure and final rMD structure. S9

SII. Supplementary figures S10-S20

Figure S1a. Fluorescence titration curve of flavonoids as a function of Pu24T concentration. S10

Figure S1b. Fluorescence titration curve of flavonoids as a function of c-kit21 and Tel-7 G-quadruplex DNA concentration. S11

Figure S2. Fluorescence titration curve of flavonoids as a function of of CT-DNA concentration. S12

Figure S3. Isothermal Titration Calorimetry **(**ITC) titration data for flavonoids binding to Pu24T at 25°C. S13

Figure S4. UV thermal denaturation profile of Pu24T G-quadruplex DNA in the absence and presence of Quercetin upto D/N = 2.0. S14

Figure S5. Expansion of NOESY spectra showing stacking interactions in base region for Quercetin and Pu24T-c-myc DNA as a function of ligand/DNA ratio upto D/N = 2.0 at 298 K. S15

Figure S6. Quercetin interacting with 5' G-tetrad of Pu24T G-quadruplex DNA via π-π stacking interactions. S16

Figure S7. Quercetin interacting with 3' G-tetrad of Pu24T G-quadruplex DNA via π-π stacking interactions S16

Figure S8. Quercetin interacting with Pu24T *c-myc* G-quadruplex DNA by formation of hydrogen bonds. S17

Figure S9. Quercetin interacting with Pu24T *c-myc* G-quadruplex DNA by stacking above the A. top G-tetrad and B. below the bottom G-tetrad. S17

Figure S10. Confoncal images monitoring morphological changes in Quercetin treated HeLa cells as compared to untreated cells. S18

Figure S11. MTT assay plot for cytotoxicity of Quercetin on HEK cells. S19

Figure S12. Effect of Quercetin at various concentrations on *c-MYC* promoter activity. S19

SI. Supplementary Tables

Table S1a. The binding constant (K_d,(M)) values of nine representative flavonoids with *c-myc* G-quadruplex DNA and CT-DNA.

**Flavonoids**	**Pu24T- *c-myc* DNA**		**CT-DNA**
	**K_d1(M)**	**K_d2 (M)**	**K_d(M)**
Luteolin	1.50×10^{-6}	8.25×10^{-5}	2.65×10^{-5}
Quercetin	1.38×10^{-7}	8.33×10^{-6}	4.26×10^{-5}
Rutin	1.71×10^{-5}	13.11×10^{-4}	7.59×10^{-5}
Kaempferol	1.30×10^{-6}	2.69×10^{-5}	3.40×10^{-6}
Puerarin	8.88×10^{-5}	8.88×10^{-4}	4.26×10^{-5}
Hesperidin	1.00×10^{-6}	9.63×10^{-5}	1.30×10^{-5}
Myricetin	1.40×10^{-6}	1.40×10^{-5}	1.60×10^{-6}
Daidzein	4.70×10^{-5}	4.70×10^{-4}	10.18×10^{-6}

Table S1b. The binding constant (K_d,(M)) values of Quercetin *c-myc* G-quadruplex DNA and c-kit21up G-quadruplex DNA

**G-quadruplex DNA**	**Quercetin**	
	**K_d1(M)**	**K_d2 (M)**
**Pu24T- *c-myc* DNA**	1.38×10^{-7}	8.33×10^{-6}
**c-ckit21up DNA**	1.12×10^{-6}	--
**Tel7 DNA**	2.13×10^{-6}	--

Table S2. Life time fluorescence decay parameters for Quercetin and its D/N = 2:1 complex with *c-myc* DNA at 298 K.

Uncomplexed Quercetin								Pu24T - c myc-Quercetin complex D/N = 2.0							
Life Time Decay (ns)			Amplitude			χ^2	Average life time (ps)	Life Time Decay (ns)			Amplitude			χ^2	Average life time (ps)
τ1	τ2	τ3	β1	β2	β3			τ1	τ2	τ3	β1	β2	β3		
5.23	62.95	7.36×10^{-2}	-3.39×10^{-2}	9.15×10^{-3}	806138.8	1.96	1.02	59.10	277.44	1.27	7.05×10^{-3}	8.40×10^{-4}	1.14	2.38	25.42

Table S3a. Thermodynamic and energetics data for the interaction Quercetin with *c-myc* DNA as obtained from ITC experiment.

Parameters	Values
N1	0.415 ± 0.05 Sites
K1	$1.57 \times 10^{7} \pm 2.14$ M^{-1}
Δ H1	-1.86 ± 284 kcal/mol
Δ S1	26.7 cal/mol/deg
N2	1.54 ± 0.14 Sites
K2	$8.50 \times 10^{5} \pm 1.54$ M^{-1}
Δ H2	-0.59 ± 179 kcal/mol
Δ S1	25.1 cal/mol/deg

Table S3b. Thermodynamic and energetics data for the interaction flavonids with *c-myc* DNA as obtained from ITC experiment.

Flavonoids	N1 (Sites)	N2 (Sites)	K1	K2
Luteolin	0.49 ± 78	0.90 ± 6.55	$13.2 \times 10^{4} \pm 3.76$ M^{-1}	0.00170 ± 2.93 M^{-1}
Rutin	0.32 ± 0.34	34.80 ± 3.27	$7.23 \times 10^{5} \pm 8.60$ M^{-1}	650 ± 8.43 M^{-1}
Kaempferol	1.15 ± 2.45	0.242 ± 160	2.09 ± 4.4 M^{-1}	$1.34 \times 10^{5} \pm 3.81$ M^{-1}
Puerarin	0.224 ± 50.4	0.432 ± 0.191	$1.10 \times 10^{4} \pm 7.75$ M^{-1}	4.48×10^{17} M^{-1}
Hesperidin	2.18 ± 4.74	0.201 ± 2.84	$7.35 \times 10^{5} \pm 1.06$ M^{-1}	$6.04 \times 10^{5} \pm 4.07$ M^{-1}
Myricetin	0.34 ± 0.285	2.05 ± 0.00	$1.65 \times 10^{5} \pm 0$ M^{-1}	$1.58 \times 10^{4} \pm 4.60$ M^{-1}
Daidzein	1.39+9.57	2.28+1.07	$3.34 \times 10^{5} \pm 1.50$ M^{-1}	$2.93 \times 10^{5} \pm 1.01$ M^{-1}

Table S4. The melting temperature values of Pu24T G-quadruplex DNA in absence and presence of Quercetin.

**D/N**	**Temperature (ºC)**
0.0	70.0
1.0	78.0
2.0	80.3

Table S5. Proton chemical shift table for Pu24T-*c-myc* DNA and Quercetin complex at D/N = 2.0.

	NH/H1	H8/H6	H1'	H2'	H2''	H3'	H71/72/73
**T1**	-	-	-	-	-	-	-
**G2**	-	8.057	-	-	-	-	-
**A3**	-	7.684	6.471	2.894	2.817	-	-
**G4**	11.758	8.014	-	-	-	-	-
**G5**	11.206	7.957	5.861	-	-	-	-
**G6**	10.920	7.894	5.761	2.481	2.641	4.048	-
**T7**	-	7.915	5.508	-	-	-	-
**G8**	11.510	7.718	6.328	-	-	-	-
**G9**	11.529	7.212	5.891	2.534	2.077	4.540	-
**T10**	-	7.392	-	-	-	-	-
**G11**	11.529	7.502	5.463	2.660	2.307	4.167	-
**A12**	-	7.555	6.150	2.627	2.875	4.240	-
**G13**	11.675	7.93	6.549	2.708	2.523	4.044	-
**G14**	11.212	7.760	5.887	2.536	2.002	4.538	-
**G15**	11.161	7.232	5.946	-	-	-	-
**T16**	-	-	-	-	-	-	-
**G17**	11.133	7.861	5.704	-	-	-	-
**G18**	11.422	7.646	5.955	2.395	2.716	4.178	-
**G19**	10.731	7.464	5.596	-	-	-	-
**G20**	10.852	7.182	5.786	-	-	-	-
**A21**	-	8.122	6.381	-	-	-	-
**A22**	-	8.151	6.205	2.345	2.522	3.542	-
**G23**	-	7.539	5.712	-	-	-	-
**G24**	11.506	7.561	5.930	-	-	-	-

Table S6. Relative intensity of intermolecular NOE connectivity between Pu24T and Quercetin in the complex at D/N=2.0 from NOESY spectra at 298K.

S.No.	NOE restraints	Input Range	Input Distance	Output Distance (2N6C)
1	A22:H8-Que:H8	m/w	3.5	4.8
2	G13:H8-Que:H8	m	4.9	5.3
3	G19:H8-Que:H8	s	4.3	5.4
4	G23:H8-Que:H8	ss	2.1	3.3
5	G2:H8-Que:H8	m	3.1	3.8
6	G6:H8-Que:H8	m	4.2	5.1
7	G17:NH-Que:H8	ss	2.8	3.5
8	G15:NH-Que:H8	s	3.0	3.6
9	G19:NH-Que:H6'	m	4.8	5.1
10	G13:H1'-Que:H6'	m	3.4	4.1
11	G24:H1'-Que:H6'	m	3.3	4.1
12	G6NH-Que:H6'	s	2.8	3.2
13	G4:H8-Que:H6'	m	3.1	3.7

Table S7. Statistics of the solution structure of Quercetin- Pu24T complex.

**NMR distance**	**Nucleic acid/ligand**
**Distance restraints**	
Total NOE	428
Intra-residue	317
Inter-residue	98
NOE- derived distance restraints	13
Hydrogen bonds	154
**Structural Statistics**<br>**Violations (mean and SD)**	
Number (> 0.2 Å)	0.0
Maximum violations (Å)	0.158 ± 0.026
Distance constraints (Å)	0.044 ± 0.019
Dihedral angle constraints (°)	0.090 ± 0.10
**Average pairwise r.m.s.d.** (Å)**	
All nucleotides	0.9976 ± 0.11
All heavy atoms	0.5964 ± 0.16

Table S8. Energy terms (kcal mol^{-1}) for starting structure and final rMD structure.

**Structure**	**Potential energy (kcal mol^{-1})**	**Van der Waals Energy (kcal mol^{-1})**	**Electrostatic Energy (kcal mol^{-1})**
**Initial**	4.46×10^{11}	1664.30	-15813.47
**Final**	-18685.50	-14891.95	-14908.78

SII. Supplementary Figures

Figure S1a. Fluorescence titration curve of all the eight flavonoids except Genistein; as a function of Pu24T-*c-myc* G-quadruplex DNA concentration. Solid lines represent fit according to the ligand binding two site saturation.

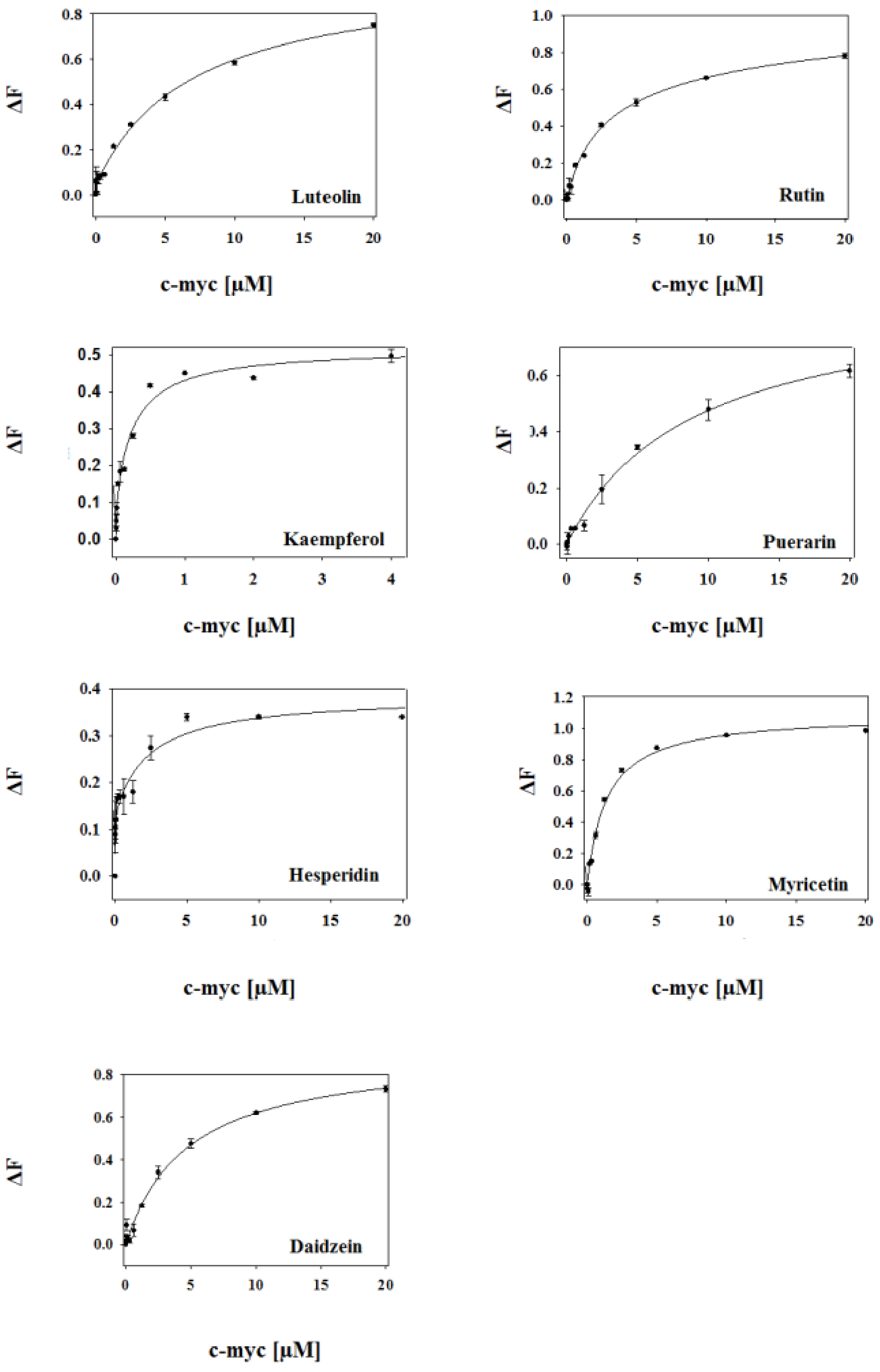

Figure S1b. Fluorescence titration curve of Quercetin as a function of: (a)c-kit21up G-quadruplex DNA concentration and (b) Tel7 (in presence of Na^+ ions) DNA concentration. Solid lines represent fit according to the ligand binding single site and two saturation.

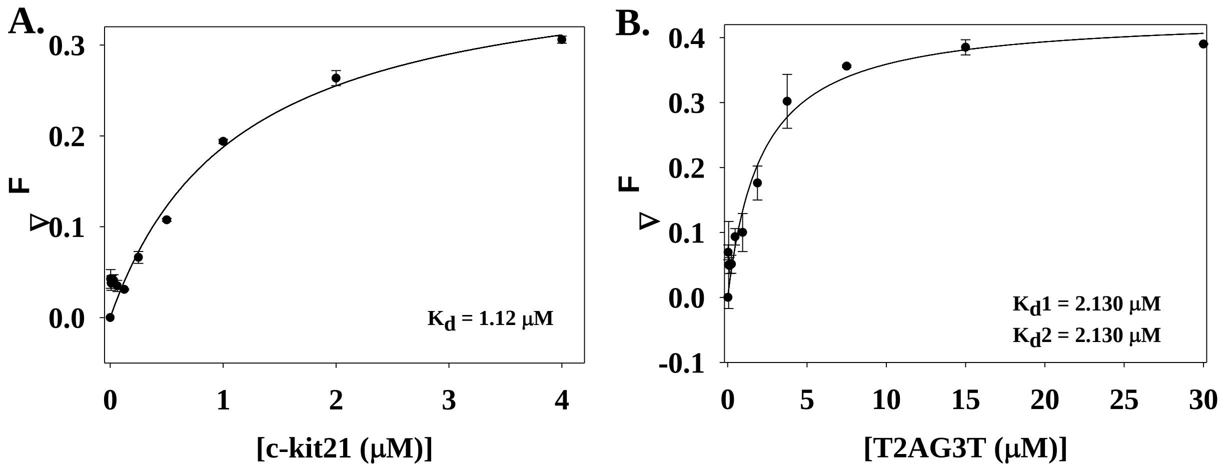

Figure S2. Fluorescence titration curve of all the eight flavonoids except Genistein; as a function of CT-DNA concentration. Solid lines represent best fit according to the ligand binding one/ two site saturation.

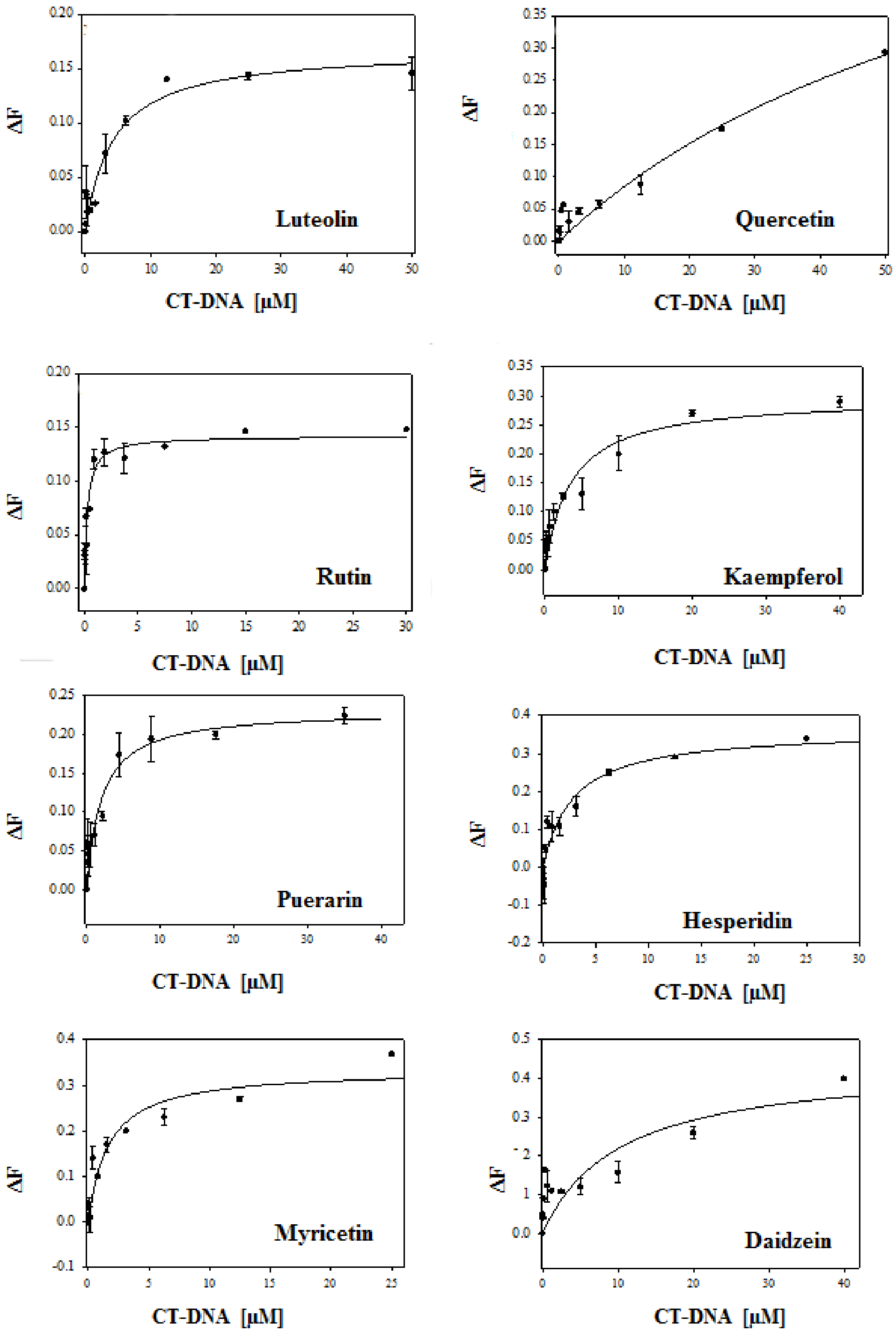

Figure S3. Isothermal Titration Calorimetry **(**ITC) titration data (points) for flavonoids binding to Pu24T at 25°C. Solid line represents the fitted data results from two site binding mode

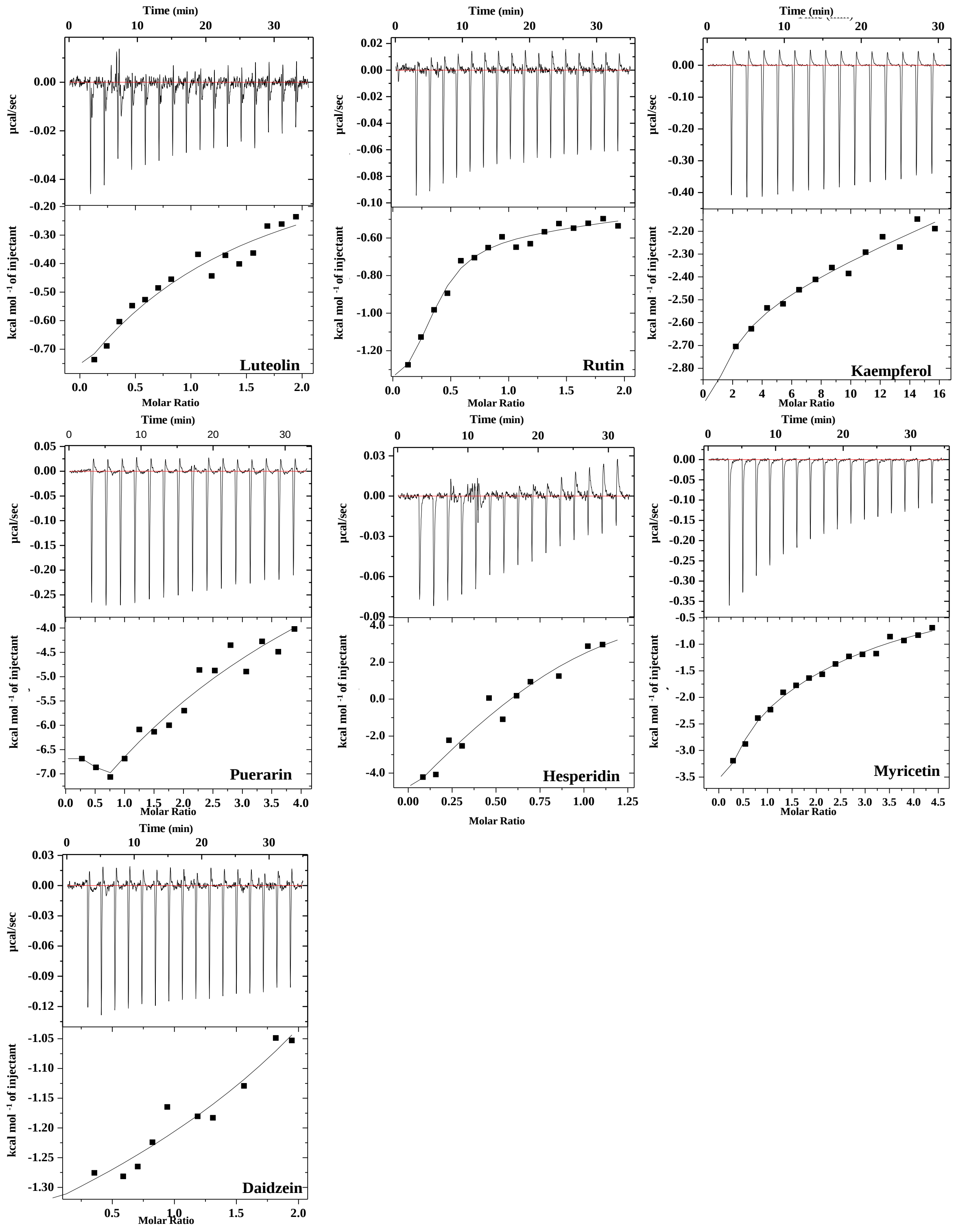

Figure S4. UV thermal denaturation profile of Pu24T G-quadruplex DNA in the absence and presence of Quercetin upto D/N = 2.0.

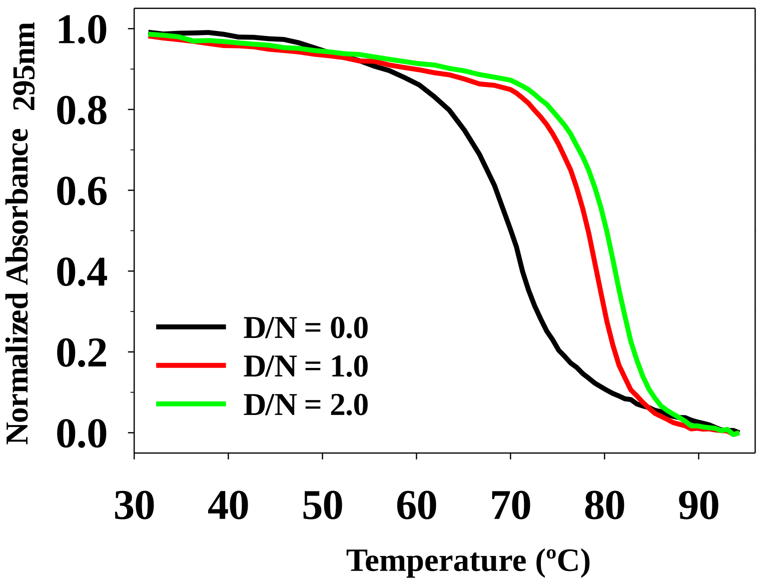

Figure S5. Expansion of NOESY spectra showing stacking interactions in base region for Quercetin and Pu24T-*c-myc* DNA as a function of ligand/DNA ratio upto **A**. D/N = 0.0 **B**. D/N = 2.0 at 298 K.

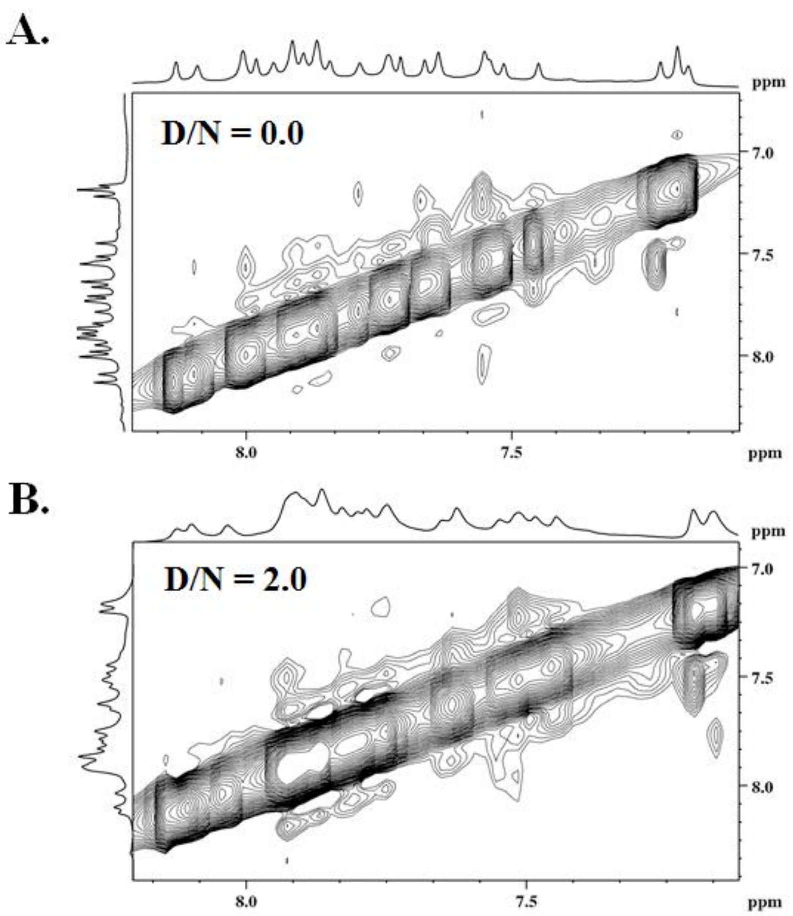

Figure S6. Quercetin interacting with Pu24T *c-myc* G-quadruplex DNA via π-π stacking interaction with 5' G-tetrad: A. top view B. side view.

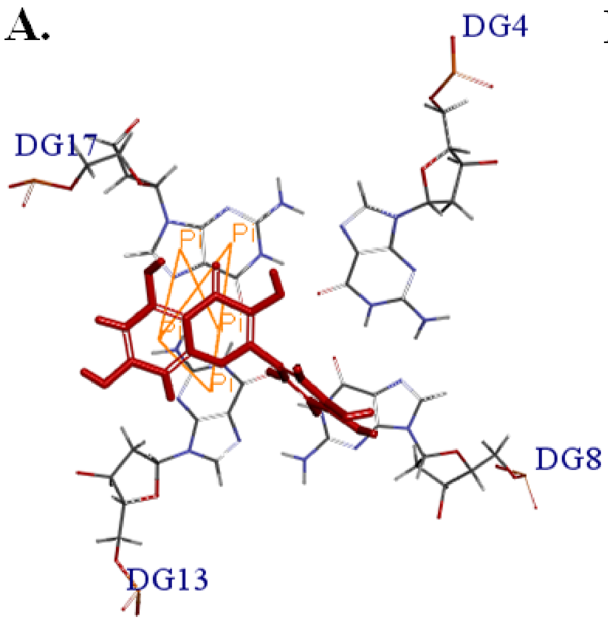

Figure S7. Quercetin interacting with Pu24T G-quadruplex DNA via π-π stacking interaction with 3' G-tetrad: A. top view B. side view.

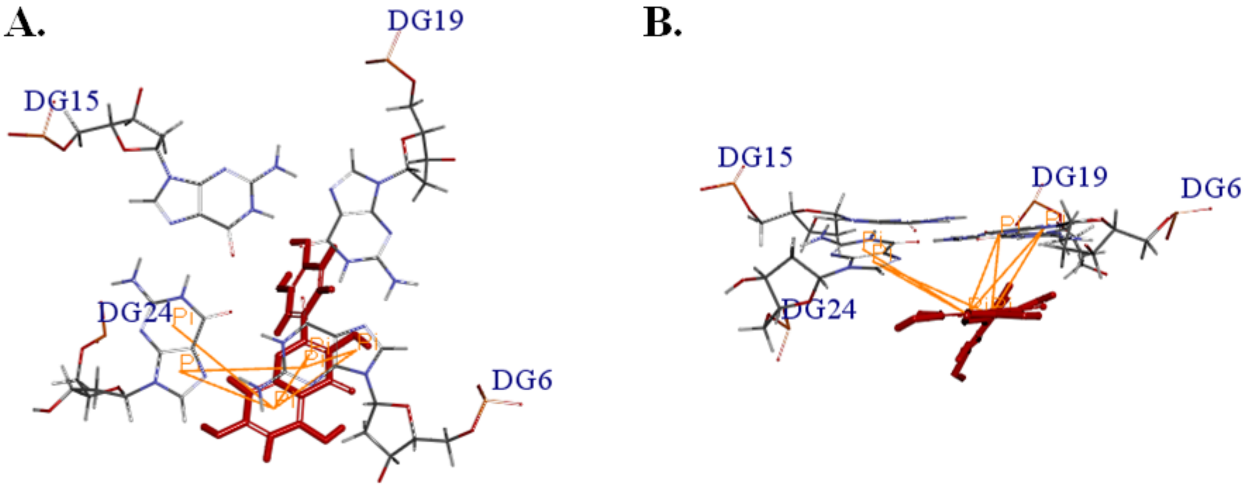

Figure S8. Quercetin interacting with Pu24T G-quadruplex DNA by formation of hydrogen bonds with: A. G-20 B. A12

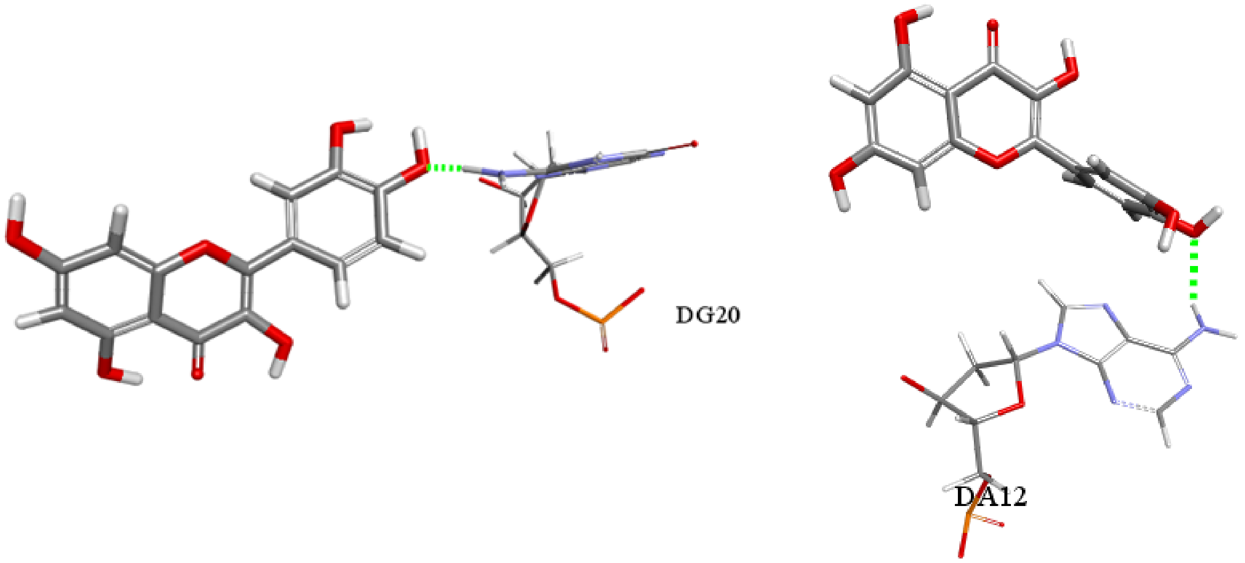

Figure S9. Quercetin interacting with Pu24T *c-myc* G-quadruplex DNA by stacking above the **A.** top G-tetrad and **B.** below the bottom G-tetrad.

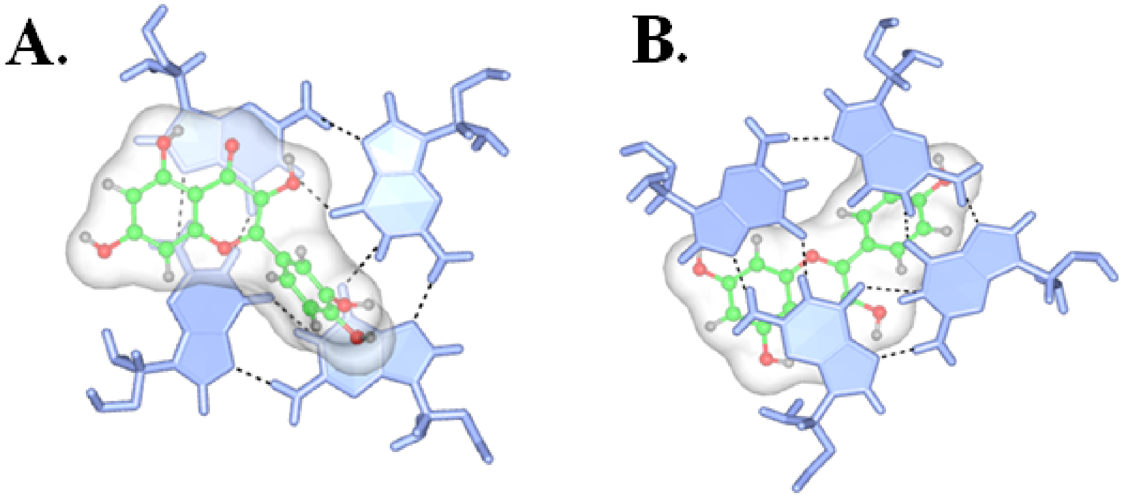

Figure S10. Upper panel shows morphological changes in Quercetin treated HeLa cells as compared to control cells monitored under phase contrast microscope. Middle panel shows confocal images of control HeLa cells stained with DAPI. Lower panel shows confocal Microscope images showing localization of Quercetin inside nucleus of the cell detected by its autofluorescence. Bottom right is the merged image of Quercetin treated cells stained with DAPI.

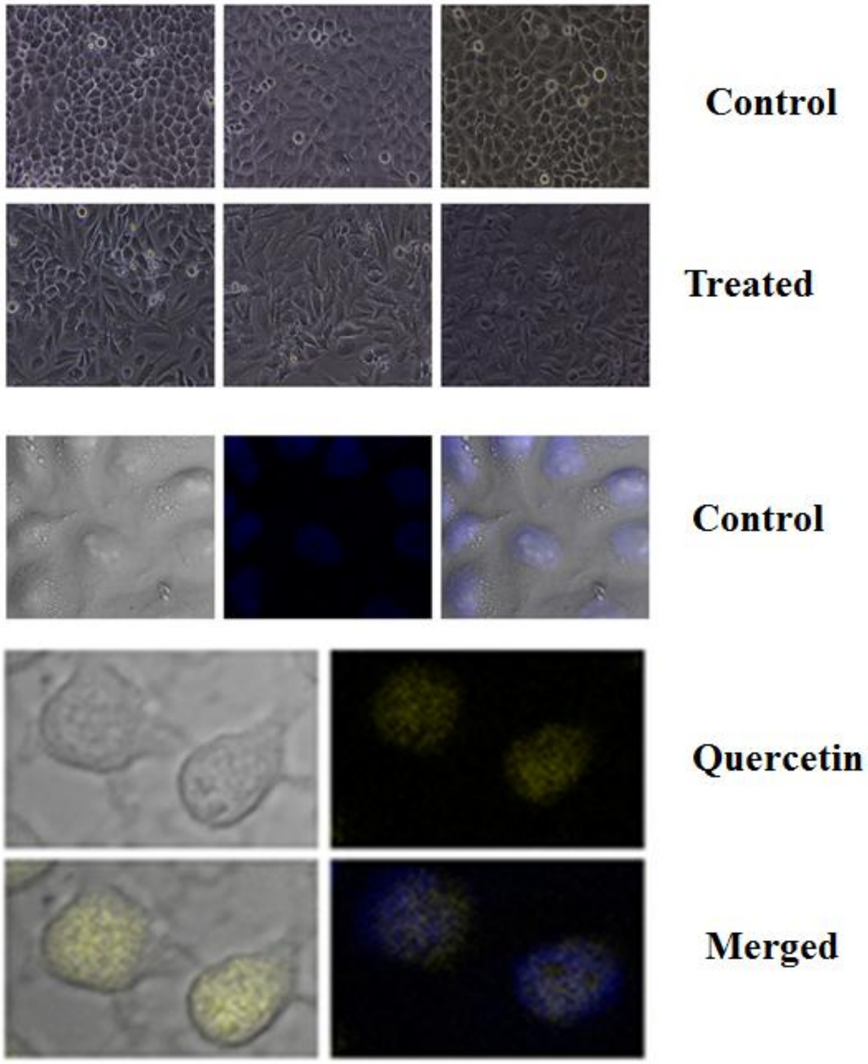

Figure S11. HEK cells were exposed to Quercetin at different concentration as indicated. Cell viability was measured by MTT assay after 48 h.

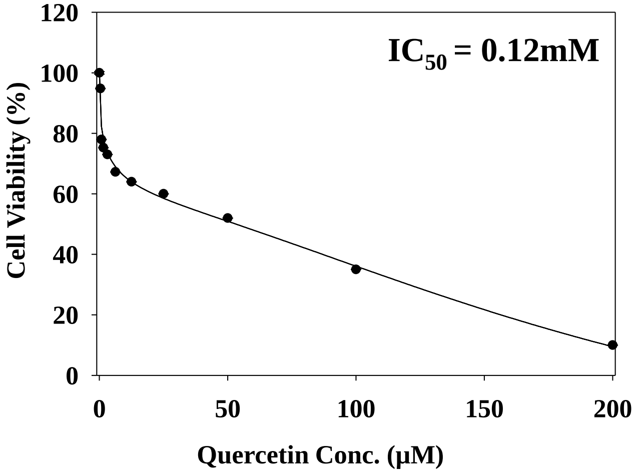

Figure S12. Effect of Quercetin at various concentrations on *c-MYC* promoter activity.

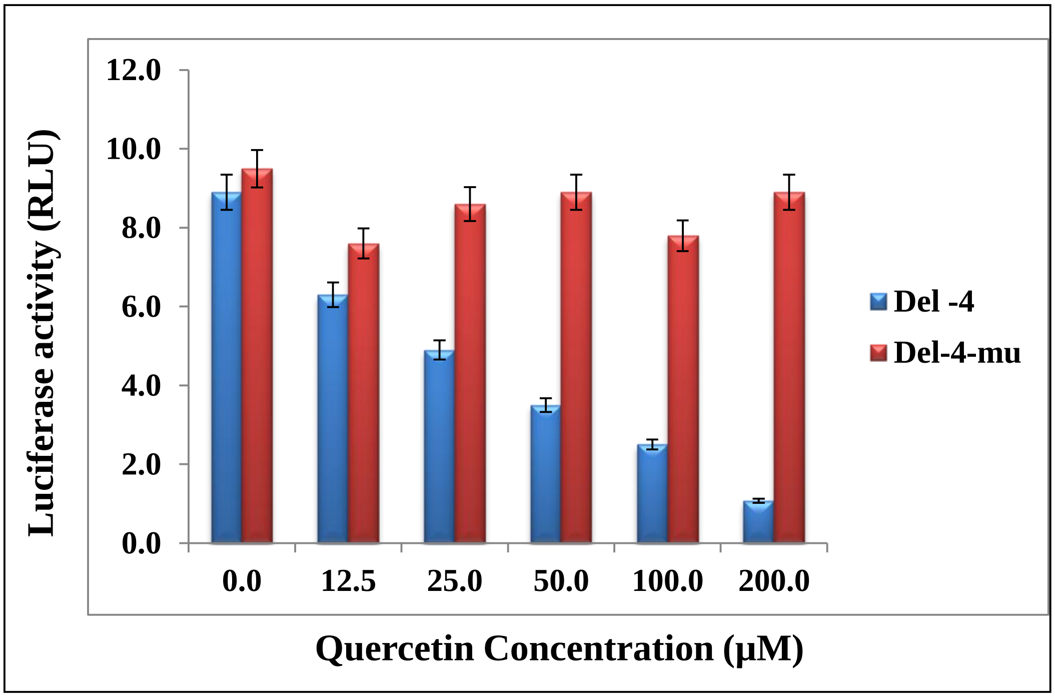

Figure S13. Representation of full gel image for semi-quantitative PCR of Quercetin treated cells

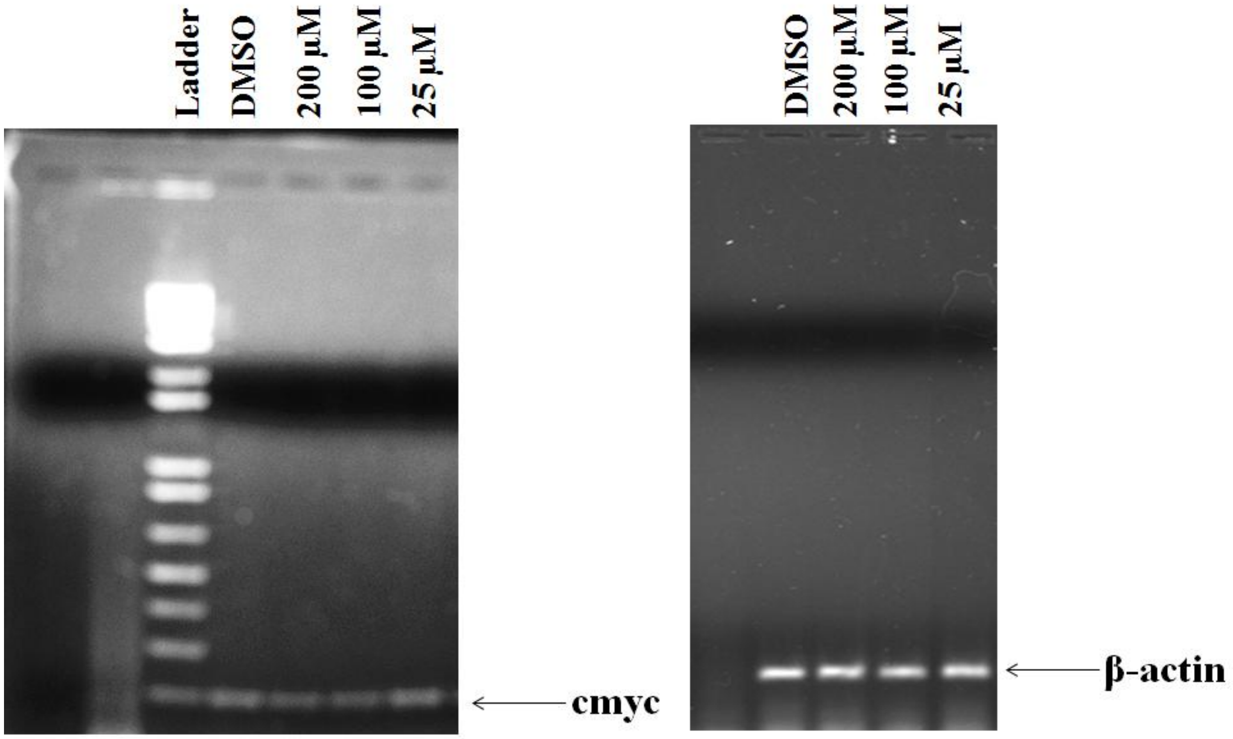

Supplement: Supplementary file 1 — Supplementary Information [file 41598_2017_3906_MOESM1_ESM.pdf]
